# Wireless Soft Athlete Bioelectronics for Monitoring Carbon Dioxide Ventilation and Physiological Performance

**DOI:** 10.1002/advs.202503880

**Published:** 2025-06-20

**Authors:** Tae Woog Kang, Ka Ram Kim, Yoon Jae Lee, Hodam Kim, Sung Hoon Lee, Youngjin Kwon, Hoon Yi, Hojoong Kim, Hyeonseok Kim, Alec Harp, Jud Ready, Melinda Millard‐Stafford, Woon‐Hong Yeo

**Affiliations:** ^1^ George W. Woodruff School of Mechanical Engineering College of Engineering Georgia Institute of Technology Atlanta GA 30332 USA; ^2^ Wearable Intelligent Systems and Healthcare Center (WISH Center) Institute for Matter and Systems Georgia Institute of Technology Atlanta GA 30332 USA; ^3^ School of Electrical and Computer Engineering Georgia Institute of Technology Atlanta GA 30332 USA; ^4^ School of Materials Science and Engineering Georgia Institute of Technology Atlanta GA 30332 USA; ^5^ School of Biological Sciences Georgia Institute of Technology Atlanta GA 30332 USA; ^6^ Electro‐Optical Systems Laboratory Georgia Tech Research Institute Atlanta GA 30332 USA; ^7^ Parker H. Petit Institute for Bioengineering and Biosciences Georgia Institute of Technology Atlanta GA 30332 USA; ^8^ Korea KIAT‐Georgia Tech Semiconductor Electronics Center (K‐GTSEC) Institute for Matter and Systems Georgia Institute of Technology Atlanta GA 30332 USA

**Keywords:** VCO_2_, athletic healthcare, flexible electronics, physiological signals, wearable device

## Abstract

Wearable devices have become essential tools for monitoring athletes' health and performance. However, most current devices are primarily designed to track physiological signals and physical indicators, which limits their ability to assess a comprehensive range of health parameters during exercise. Therefore, there is a need for a multimodal wearable platform that can capture diverse exercise‐related metrics. Here, a wireless soft athlete bioelectronic system is presented that monitors various physiological signals and metabolites during exercise. This system includes a smart‐sensing lip guard that detects ventilated carbon dioxide, osmolality, environmental conditions, and humidity—key indicators of the metabolites expelled from the nose and mouth. Additionally, the cardiac patch complements the lip guard by monitoring multiple signals, such as electrocardiograms, heart rates, temperature, and motion. This integrated system is capable of detecting exercise‐induced conditions such as dehydration, hyperventilation, and abnormal heart signals. By combining data from both physiological and environmental factors, the wearable device offers a comprehensive assessment of how external conditions influence an athlete's performance. Evaluating the device with human subjects under varying temperature conditions demonstrates its effectiveness in observing exercise performance, regardless of environmental factors, highlighting its significant potential for enhancing athlete healthcare and performance monitoring.

## Graphical abstract

A wireless soft athlete bioelectronic system offers a real‐time continuous monitoring of various physiological signals and metabolites during exercise.

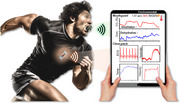

## 1 Introduction

Advances in wearable devices monitor various health‐related parameters with integrated multimodal sensors,^[^ ^1^, ^2^, ^3^ ^]^ including motion and temperature indicators,^[^ ^4^, ^5^ ^]^ electrophysiological signals,^[^ ^6^, ^7^, ^8^, ^9^ ^]^ and biomarkers.^[^ ^6^, ^10^, ^11^ ^]^ These devices have broadened their applications from clinical health monitoring to daily well‐being,^[^ ^12^, ^13^, ^14^ ^]^ enabling continuous tracking of sleep,^[^ ^15^, ^16^, ^17^ ^]^ exercise,^[^ ^3^, ^18^ ^]^ and stress.^[^ ^9^, ^19^, ^20^, ^21^ ^]^ Among them, monitoring health parameters during exercise helps identify athletes` health indicators, assess their condition and performance,^[^ ^22^, ^23^ ^]^ and prevent potential health risks and accidents from physical stress.^[^ ^24^, ^25^ ^]^ These parameters have been reported to be affected not only by individual mental and physical conditions but also by environmental humidity and temperature.^[^ ^26^, ^27^, ^28^ ^]^ High temperature and humidity induce higher heart rates and dehydration during exercise, and they cause heat‐related strokes.^[^ ^29^, ^30^, ^31^ ^]^ Therefore, indices such as the heat index and wet‐bulb globe temperature are widely used in sports and industrial health to assessheat stress and guide safe activity levels under hot and humid conditions.^[^ ^32^, ^33^ ^]^ However, currently developed multimodal wearable devices are typically based on electrophysiological signals such as electrocardiogram (ECG),^[^ ^2^, ^34^, ^35^ ^]^ electromyogram (EMG),^[^ ^36^, ^37^, ^38^, ^39^ ^]^ and electroencephalogram (EEG),^[^ ^15^, ^40^ ^]^ and other physical indicators, including inertial measurement unit (IMU), and body temperature. So, they are still required to use additional instruments for accurate athlete performance monitoring and to prevent risks. Meanwhile, athletes release a lot of metabolites such as sweat, saliva, and carbon dioxide (CO_2_) during exercise; several sensors have been reported to detect them, but they are still under development for single‐modal device platforms.^[^ ^41^, ^42^, ^43^, ^44^ ^]^ Therefore, it is necessary to develop a highly multimodal wearable device that includes not only traditional parameters like physiological signals and indicators but also environmental parameters and metabolites to monitor athletes` healthcare and performance monitoring.

The human body maintains homeostasis during the daily cycle.^[^ ^45^, ^46^ ^]^ When humans do an activity, the body consumes oxygen and nutrients,^[^ ^47^, ^48^ ^]^ releasing CO_2_ and metabolic waste products.^[^ ^49^, ^50^, ^51^ ^]^ These byproducts are removed from the body through physiological processes, such as breathing, sweating, and urination. However, in the case of rigorous activity such as exercise, the energy consumption rate increases significantly, making it difficult to maintain homeostasis and leading to an imbalance in the body.^[^ ^52^, ^53^, ^54^, ^55^ ^]^ This phenomenon causes dehydration,^[^ ^56^, ^57^, ^58^, ^59^ ^]^ making the body feel thirsty. And it decreases activity efficiency with dizziness and fatigue,^[^ ^60^, ^61^, ^62^ ^]^ and severe dehydration can lead to shock or even death.^[^ ^58^, ^63^, ^64^ ^]^ In particular, environmental factors such as temperature, humidity, and pressure can significantly disrupt homeostasis by increasing thermal strain and fluid loss, resulting in accelerated dehydration.^[^ ^28^, ^65^ ^]^ Traditionally, dehydration has been analyzed using blood tests, urinary specific gravity, and body mass measurement.^[^ ^66^, ^67^ ^]^ Although blood tests are known as the gold standard method for dehydration, they are invasive and time‐consuming for preparation steps.^[^ ^68^ ^]^ Urinary specific gravity and body mass measurements are non‐invasive tools for dehydration; however, they are still unsuitable for exercise since they cannot be conducted in real time during the exercise.^[^ ^69^ ^]^ To address this, it has been recently reported that salivary measurement can be considered an indirect biomarker for dehydration monitoring without obstructing exercise.^[^ ^43^, ^70^, ^71^ ^]^ Previously, our research indicated that salivary osmolality can be correlated with heart rate variability during exercise, but it remains to be studied whether salivary osmolality is affected by environmental changes, as traditional measurements are affected.

Arterial blood gases (ABG) carry important health information about the pulmonary gas exchange, and they have been commonly used in intensive care as a diagnostic tool to assess the function of the cardiorespiratory system.^[^ ^72^, ^73^ ^]^ The ABG test evaluates the blood oxygenation and acid‐base balance by measuring the partial pressure of oxygen (PaO_2_),^[^ ^74^ ^]^ partial pressure of carbon dioxide (PaCO_2_),^[^ ^75^ ^]^ and pH in arterial blood.^[^ ^76^ ^]^ Among them, PaCO₂ is a critical parameter for assessing respiratory function, indicating exercise‐induced physiological abnormalities such as hypercapnia or hyperventilation, which are significantly associated with reduced performance, dizziness, or even early signs of heat stress.^[^ ^77^, ^78^, ^79^ ^]^ However, even though ABG is considered the gold standard method, the ABG test is invasive, limited to central hospitals, and unable to provide continuous measurement,^[^ ^80^, ^81^ ^]^ which makes it unsuitable for tracking outdoor activity, such as exercise. To enable non‐invasive and real‐time blood gas monitoring, alternative approaches such as transcutaneous blood gas measurement and tidal gas analysis hold significant potential for continuous and dynamic monitoring.^[^ ^82^, ^83^, ^84^, ^85^ ^]^ Transcutaneous blood gas measurement analyzes diffused gas from the blood vessel to the skin.^[^ ^82^, ^86^ ^]^ However, heating the skin is required to help gas diffusion,^[^ ^87^ ^]^ so it can be easily affected by body and environmental temperature variables. Also, they need to use a closed chamber to store diffused gases between the skin and the sensor, making them highly sensitive to individuals` motion and humidity.^[^ ^88^ ^]^ For this reason, transcutaneous blood gas often shows delayed and broad responses. Tidal gas measurement from exhaled breath is another potential method for continuously monitoring blood gas. However, currently developed tidal sensors are designed with a wired connection; wireless and real‐time measurement devices should be developed for outdoor exercise monitoring.^[^ ^89^, ^90^, ^91^ ^]^

Here, this study introduces an all‐in‐one smart‐sensing lip guard and soft cardiac patch to monitor health parameters from the face and chest. The smart‐sensing lip guard measures ventilated CO_2_ (VCO_2_) and saliva osmolality, which are important metabolism signals related to exercise performance and healthcare. The smart‐sensing lip guard is also integrated with an environmental humidity sensor, which measures ambient pressure, temperature, and humidity, enabling athletic performance monitoring under different environmental conditions. The soft cardiac patch measures physiological signals such as electrocardiograms, heart rate, body temperature, acceleration, and gyroscope. With its high multimodality, it can measure a broad range of signals from the face and chest during exercise. Both devices can also be connected wirelessly to portable devices such as tablets or cell phones and deliver their signals simultaneously. The smart‐sensing lip guard and soft cardiac patch are designed with flexible printed circuit boards and sensors, allowing them to conform seamlessly to the human skin or another material surface.^[^ ^92^ ^]^ The smart‐sensing lip guard was validated with commercial salivary dehydration measurement devices. These all‐in‐one devices were also assessed under different environmental conditions and showed the high potential of wearable devices in exercise performance and healthcare monitoring.

## 2 Results and Discussion

### 2.1 Overview of an All‐In‐One Wearable Multimodal Sensing System

**Figure 1:**
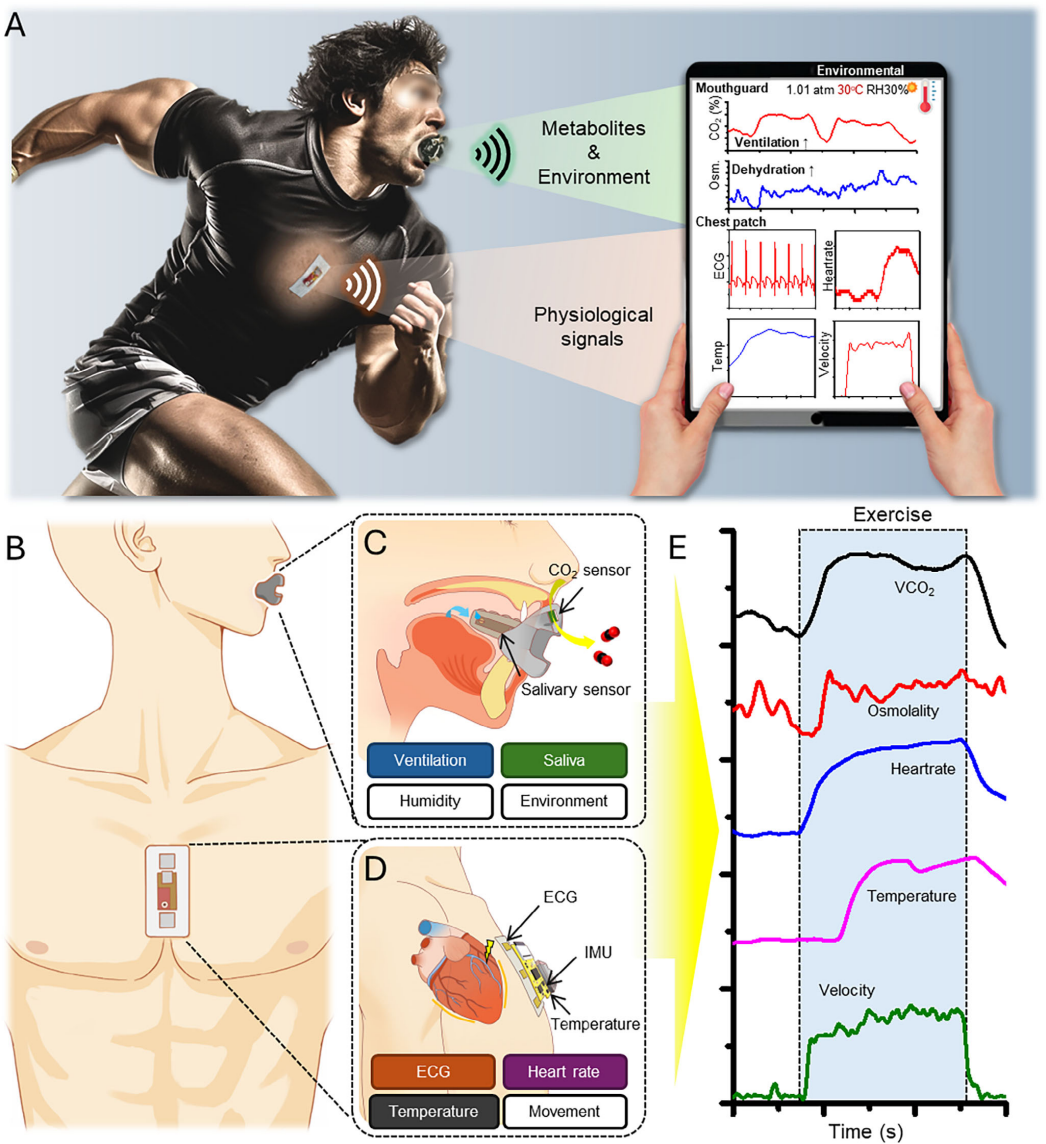
Overview of all‐in‐one wearable devices for exercise performance monitoring and healthcare. A) Illustration of the all‐in‐one smart‐sensing lip guard and soft cardiac patch, along with a tablet capturing multiple wireless signals from those devices in real time. B) Locations of the wearable devices: one in the mouth and the other on the sternum. C, D) Illustration showing multiple sensors of the smart‐sensing lip guard (C) and soft cardiac patch (D). E) Measured signals from the smart‐sensing lip guard and soft cardiac patch during exercise, capturing changes of VCO_2_, osmolarity, heart rate, temperature, and velocity.

**Figure 2:**
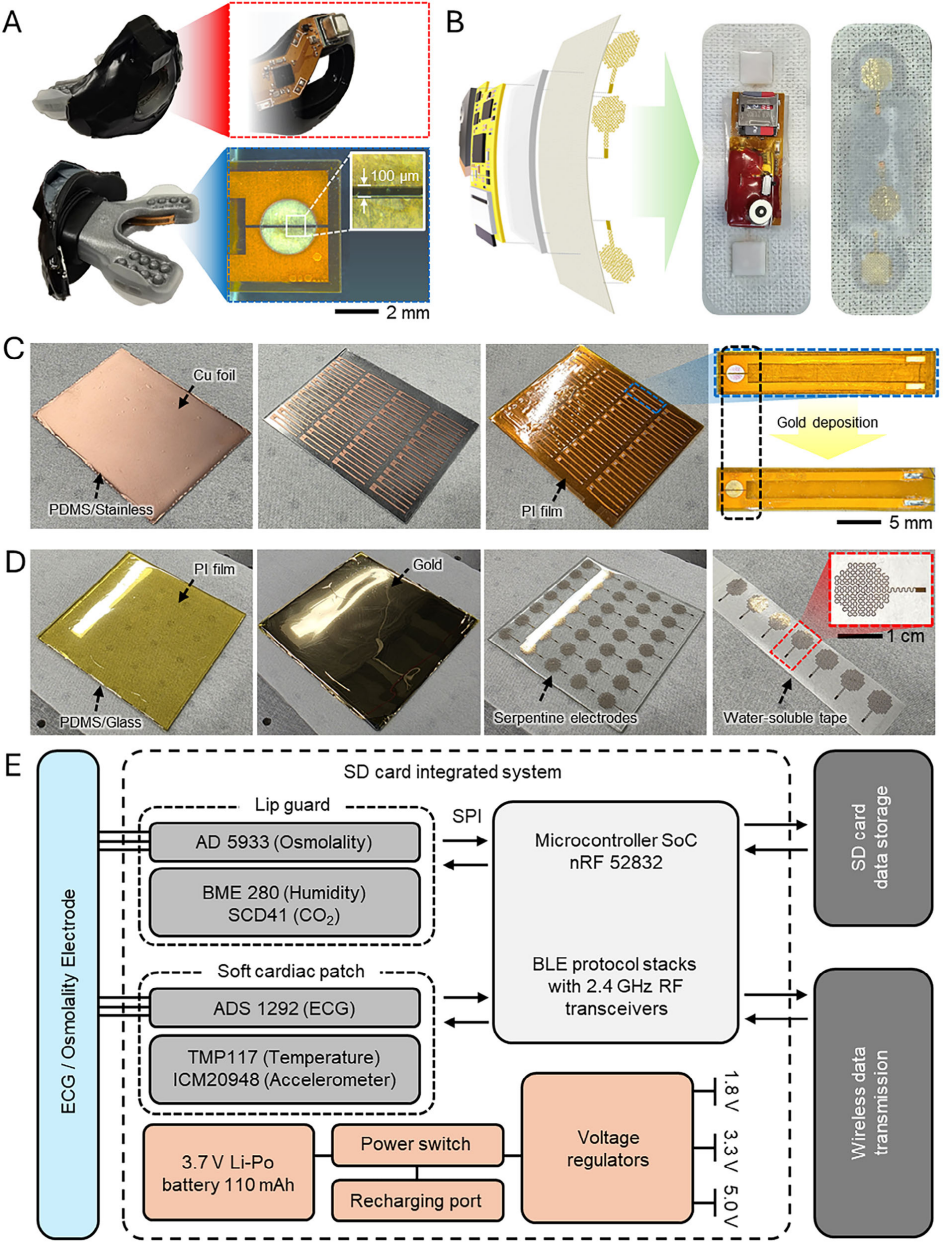
Materials, structures, and design of the integrated system. A) Photo of the smart‐sensing lip guard, including a humidity sensor, CO_2_ sensor, and osmolality sensor. B) Photo of the soft cardiac patch that measures ECG, temperature, and movements. C) Gold capacitance sensor in the lip guard for salivary osmolality measurement. D) Gold serpentine electrode in the cardiac patch for ECG measurement. E) Flow chart showing the overview of the system components and data acquisition using the smart‐sensing lip guard and soft cardiac patch.

This paper introduces an all‐in‐one integrated wearable system that has a smart‐sensing lip guard and a cardiac patch tomeasure physiological signals for their healthcare and performance monitoring during exercise (**Figure** **1**A). The devices feature wearable and wireless communication, delivering signals in real time. Each device is placed on the mouth and cardiac center (Figure 1B). A smart‐sensing lip guard was developed to measure salivary and respiratory activity during exercise (Figure 1C). The smart‐sensing lip guard integrates an admittance osmolality sensor, a carbon dioxide sensor, and an environmental humidity sensor. Those sensors detect various signals from the mouth and nose of an athlete, which can be analyzed to determine exercise performance. The admittance osmolality sensor continuously measures salivary osmolality, a key indicator of dehydration.^[^ ^3^, ^93^ ^]^ The carbon dioxide sensor, integrated into the lip guard with a channel, collects exhaled breath from the nose and mouth to measure VCO_2_, humidity, and temperature. This functionality is essential for monitoring hypercapnia, hyperventilation, and lung dysfunction. Compared with previously reported wearable CO_2_ monitoring devices, the smart‐sensing lip guard represents high multimodality with a wireless connection, reproducibility, and long‐lasting performance for daily activity monitoring (Table S1, Supporting Information). The environmental humidity sensor is placed outside of the smart‐sensing lip guard, and it measures ambient atmospheric air pressure, humidity, and temperature. This capability makes the device useful for monitoring athletic performance in various environmental conditions, such as summer and winter or alpine and desert settings. In addition to the smart‐sensing lip guard, the soft cardiac patch measures ECG, body skin temperature, acceleration, and gyroscope (Figure 1D).^[^ ^3^, ^8^, ^94^ ^]^ Also, the soft cardiac patch measures heart rate derived from ECG and velocity from acceleration. The wearable smart‐sensing lip guard and soft cardiac patch feature an all‐in‐one multimodal measurement system for many physiological signals during exercise: environmental atmosphere, environmental temperature, ambient humidity, ventilated carbon dioxide, breath humidity, breath temperature, salivary osmolality, ECG, heart rate, body temperature, acceleration, gyroscope, and velocity (Figure 1E) to monitor the athletic performance and healthcare from the nose, mouth, and chest of the participants. The smart‐sensing lip guard was designed with a flexible printed circuit board (fPCB) device (Figure S1, Supporting Information) and integrated into a commercialized lip guard already validated as medical‐grade for human application. **Figure** **2**A shows the front and rear views of the smart‐sensing lip guard. Due to its flexibility, the circuit can be seamlessly placed on the curved surface of the lip guard, ensuring a conformal fit (Figure S2, Supporting Information). A CO_2_ sensor was placed in the 3‐dimensional (3D)‐printed channel to detect ventilation (Figure S3, Supporting Information). The channel was designed as a conversing structure, which had two holes on top and bottom to collect breath from the nose and mouth. An environmental humidity sensor was placed outside the channel to detect ambient air conditions. This 3D‐printed channel helps not only collect VCO_2_ from the inside CO_2_ sensor but also separates the BME280 humidity sensor from the exhaled gas (Figure S4, Supporting Information). The admittance osmolality sensor was placed on the side of the lip guard. The sensor had a 100 µm gap between the electrodes, and it measured changes in admittance caused by electrolytes when saliva came into contact with the sensor. The wearable soft cardiac patch was designed with medical‐grade acrylate adhesive for one‐time use (Figure 2B). A silicone damping layer is positioned between the circuit and the adhesive tape to reduce strain on the circuit and create a conformal fit to the chest. It also gave an advantage to the circuit, which could be recycled with easy separation from the patch. Sensors for osmolality and ECG electrodes are directly in contact with the human skin (Figure 2C).^[^ ^3^ ^]^ To give biocompatibility, sensor edges were covered with polyimide tape, and gold was reductively deposited on the contact tip area (Figure S5, Supporting Information). The ECG electrode was fabricated using a microfabrication process (Figure S6, Supporting Information), where a 200 nm gold layer was deposited onto a polyimide (PI) film via e‐beam evaporation. The electrode was then patterned into a serpentine structure to provide flexibility and stretchability (Figure 2D; Figure S7, Supporting Information).^[^ ^15^, ^95^ ^]^ Both osmolality and ECG electrodes could be mass‐produced, which is beneficial for disposable use. Figure 2E describes the flow chart of the wearable soft cardiac patch and smart‐sensing lip guard. Signals were detected from the nose, mouth, and chest by electrodes and sensor chips, and they were delivered to the microcontroller system on chip (SoC). SoC then generated a Bluetooth signal and communicated with mobile devices such as tablets or cellphones. Also, it was integrated with an SD card storage system, and all data could be saved and read afterward with a 99.5% retention rate (Figure S8, Supporting Information).

### 2.2 Characterization of Sensing Properties of the Wearable Smart‐Sensing Lip Guard

**Figure 3:**
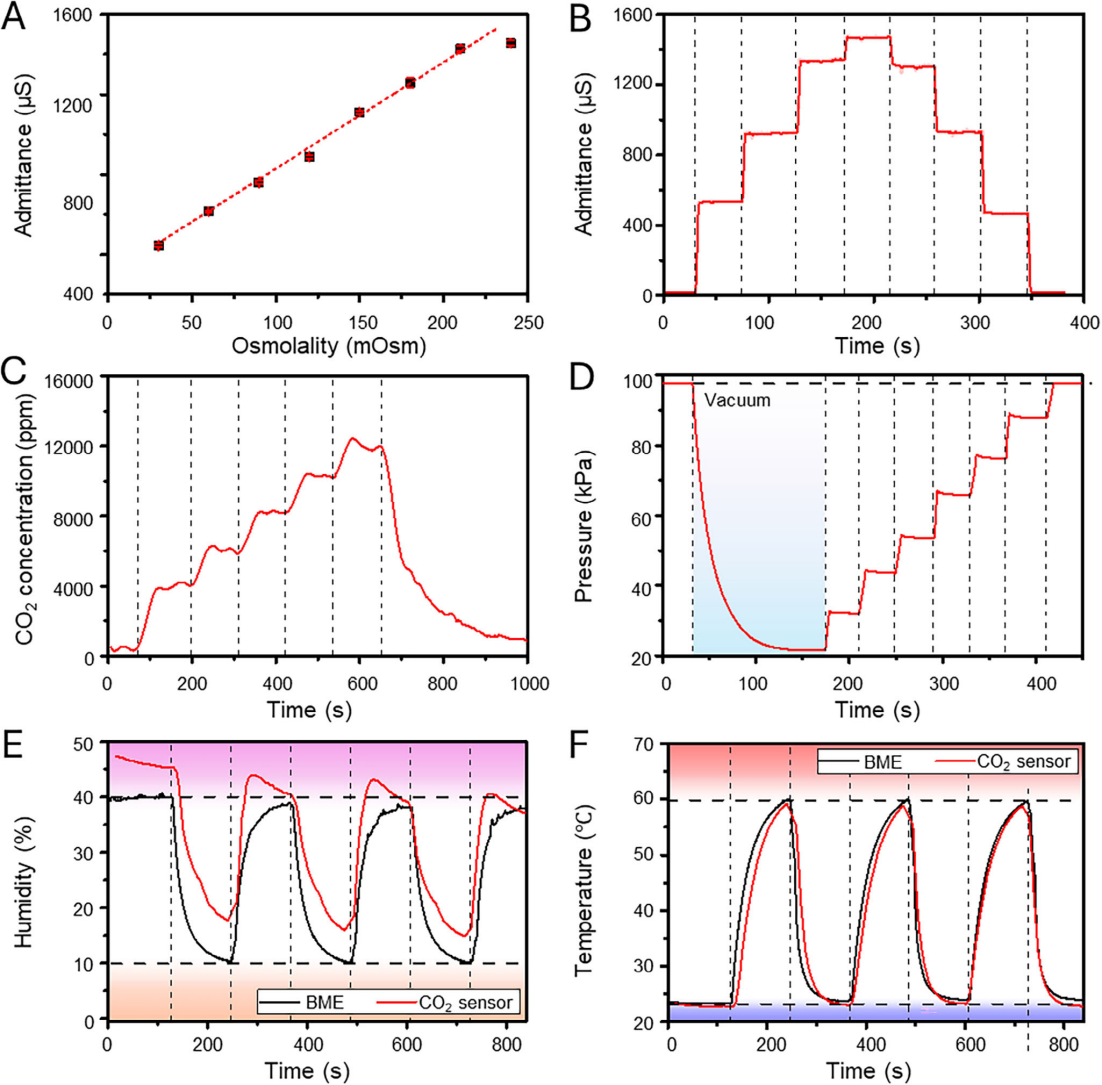
Characterization of the smart‐sensing lip guard. A) Calibration of the osmolality sensor. B) Continuous osmolality detection under various artificial saliva with different electrolyte concentrations. C) Continuous CO_2_ measurements with different concentrations from 0 to 12 000 ppm under ambient atmosphere. D) Environmental pressure measurement using a humidity sensor from vacuum to atmosphere (100 kPa). E) Dynamic humidity monitoring of both BME (humidity) and CO_2_ sensors with ambient conditions and in the oven, and F) temperature monitoring during the measurement.

The sensors on the smart‐sensing lip guard were evaluated to confirm their accuracy, reliability, and overall functionality. To characterize the osmolality sensor, electrochemical impedance spectroscopy (EIS) was performed using artificial electrolyte concentrations ranging from 30 to 240 mOsm, over a frequency range of 100 to 100 Hz. Figure S9 (Supporting Information) shows the sensor responses between 100 and 10 kHz, thereby excluding the polarization effect and non‐ideal capacitive behavior at low frequencies.^[^ ^96^, ^97^ ^]^ Each curve exhibited concentration‐dependent changes in the real component of impedance.^[^ ^98^ ^]^ The osmolality sensor was then calibrated and characterized by measuring its response to varying concentrations of artificial electrolytes (**Figure** **3**A). The sensor showed linear admittance responses with a high coefficient of determination (R^2^ = 0.997). The sensor could respond significantly to concentration change, even if the concentration was decreased (Figure 3B; Figure S10, Supporting Information). For its stability test, the admittance sensor was measured for continuous signal measurement dipping in random electrolyte concentrations among 60, 120, and 210 mOsm (Figure S11, Supporting Information). The sensor showed a reversible response with repeated dipping. The sensor was also assessed for long‐term stability by dipping it in electrolytes for 6 days, and it showed excellent stability with a negligible signal decrease of 5 days (Figure S12, Supporting Information). To validate the CO_2_ sensor response, it was placed into the closed chamber and characterized by different concentrations of CO_2_ (Figure 3C). The sensor showed great responses with discrete increments in CO_2_ concentration. When the CO_2_ was removed from the chamber, the sensor showed a reliable response under decreasing concentrations. The environmental atmosphere of the humidity sensor was characterized by a vacuum pump. As shown in Figure 3D, the sensor quickly responded under vacuum and increased stepwise with discrete pressure control. The humidity and temperature responses of the lip guard were validated with a dry oven (Figure 3E,F). The device was placed in the dry oven (60 °C, 10%) and then put out of the oven (23.6 °C, 40%) for 2 min for each step (Figure S13, Supporting Information). The environmental humidity sensor responded quickly under varying conditions with repeated steps. CO_2_ sensors also showed reliable responses. However, it seemed delayed under varying conditions because of its sensor mechanism, which measures changes in the sensor so that it can be affected by the diffusion of the molecule. CO_2_ sensor. Both temperature responses showed reliability with each other under several repetitions.

### 2.3 Validation of the Wearable Soft Cardiac Patch During Exercise

**Figure 4:**
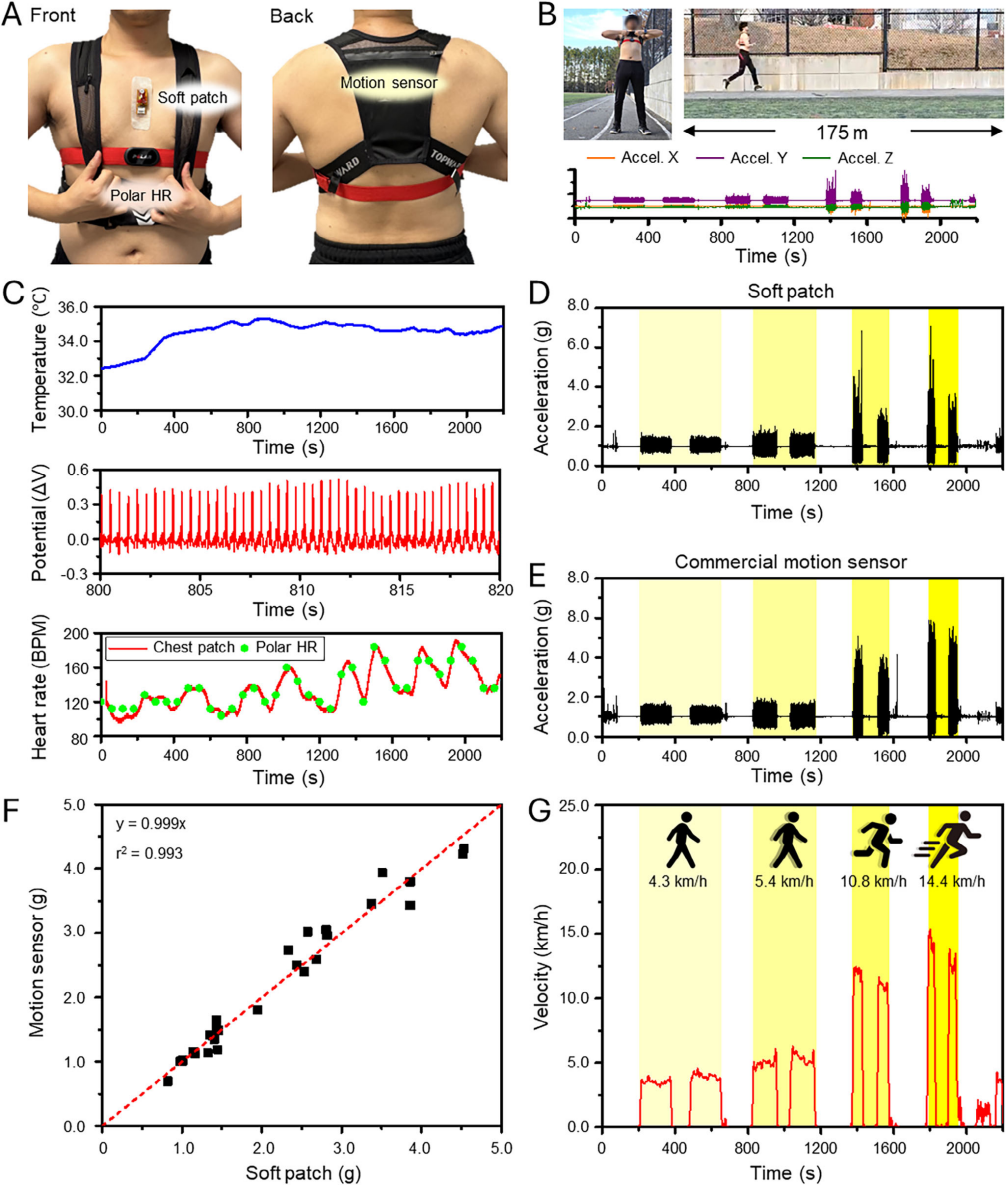
Performance demonstration of the soft cardiac patch. A) Photos of a subject wearing the soft cardiac patch, a commercial heart rate sensor (Polar HR), and a commercial motion sensor. B) Photos of the exercise test and the measured acceleration responses during the exercise. C) Measured data during the exercise, including temperature, ECG, and heart rate signals. D, E) Comparison of measured movement signals between the wearable soft patch (D) and the commercial motion sensor (E). F) Correlation analysis of the data in (D‐E), showing a high linear relationship between the two devices. G) Assessment of the measured velocity from the soft cardiac patch during different running conditions.

After the characterization of the sensing property, the wearable soft cardiac patch and smart‐sensing lip guard were worn by a subject and validated during activity. First, the soft cardiac patch was validated with commercial devices. As shown in **Figure** **4**A, a subject wore the soft cardiac patch, HR meter (Polar HR), and motion sensor (LSM6DSV). The subject exercised on 175 m of track with different states from walking (4.3 km h^−1^), fast walking (5.4 km h^−1^), jogging (10.8 km h^−1^), and running (14.4 km h^−1^) (Figure 4B). During these activities, the soft cardiac patch successfully recorded increased body temperature and detailed ECG waveforms, as illustrated in Figure 4C. Heart rate was calculated from the ECG signal, and it showed fluctuation due to running and a higher heart rate with fast exercise. The heart rate of the soft cardiac patch was compared with the heart rate meter, and similar trends were observed. During exercise, the acceleration of the soft cardiac patch and the motion sensor were measured. Figure 4D shows the total acceleration of the soft cardiac patch. It showed different oscillation frequencies and g‐forces with different speeds. At the same time, motion sensors measured acceleration during running (Figure 4E). From both devices, 30 points of acceleration were randomly chosen and compared, and they showed quite linear properties with a slope of 0.999 and a high R^2^ (0.993), which means the soft cardiac patch could measure exact acceleration (Figure 4F). Additionally, the velocity of the subject could be measured by integration of the acceleration. Figure 4G showed different velocities during running. Each estimated speed was similar to the practical speed.

### 2.4 Performance Validation of Devices During Exercise

**Figure 5:**
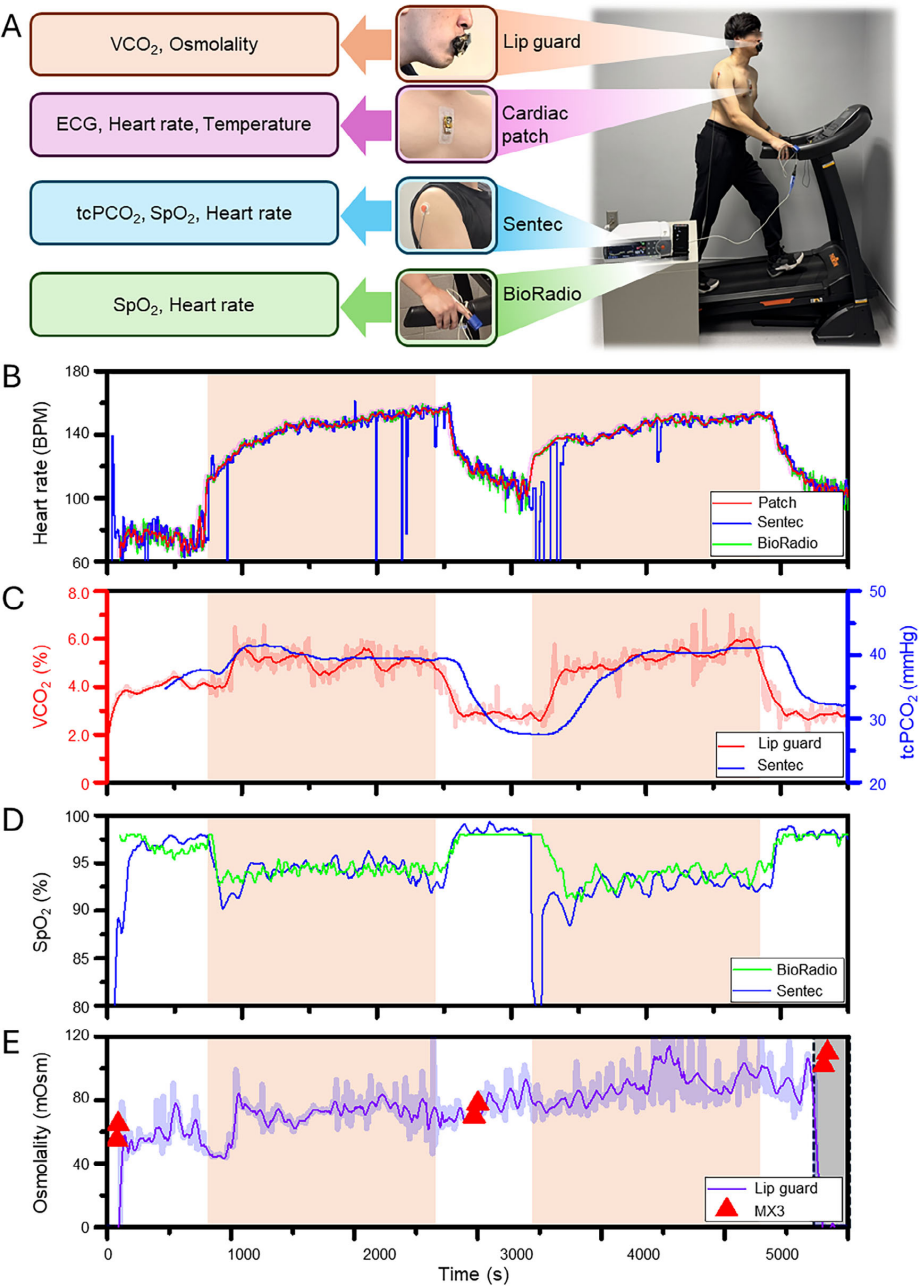
Performance validation of the lip guard and the cardiac patch compared to commercial devices during indoor treadmill exercise. A) Multiple target signals measured by the lip guard, soft cardiac patch, and commercial devices (Sentec and BioRadio) during an exercise with a treadmill. B) Comparison of measured heart rate signals between the wearable patch, Sentec, and BioRadio. C) Comparison of CO_2_ responses between VCO_2_ from the smart‐sensing lip guard and tcPCO_2_ from Sentec. D) Comparison of SpO_2_ values between Sentec and BioRadio for reliability confirmation of the tcPCO_2_ measurement. E) Comparison of osmolality from the smart‐sensing lip guard and hydration values from a point‐contact commercial device (MX3).

The smart‐sensing lip guard's CO_2_ and osmolality sensors were then validated against commercial benchmarks, including the transcutaneous partial carbon dioxide (tcPCO_2_) sensor (Sentec),^[^ ^82^, ^90^, ^99^ ^]^ oxygen saturation (SpO_2_) monitor (BioRadio),^[^ ^100^ ^]^ and dehydration meter (MX3).^[^ ^3^, ^101^ ^]^ Results confirmed its reliability for continuous, real‐time exercise monitoring. **Figure** **5**A shows a subject who wore the whole suite of sensing devices. The subjects wore the smart‐sensing lip guard and the soft cardiac patch on their mouth and chest; the nIR sensor for Sentec was placed on their deltoid, and the BioRadio sensor was placed on their index finger. Unlike the validation test of the soft cardiac patch, those sensors are sensitive to motion. So, a mild treadmill condition with a 15° angle and 4.8 km h^−1^ was used for measurement. Sentec could measure tcPCO_2_, SpO_2_, and heart rate; BioRadio could measure SpO_2_ and heart rate. So, this comparison method could ensure double validation for the lip guard. MX3 measured osmolality before and after exercise and during rest. Figure 5B shows the heart rate of the soft cardiac patch, Sentec, and BioRadio during exercise. While Sentec exhibited some noise due to motion artifacts, the overall trends were consistent with the data from the soft cardiac patch and BioRadio (Figure S14, Supporting Information). Before comparing CO₂ trends between the smart‐sensing lip guard and Sentec, it was confirmed that the smart‐sensing lip guard's CO₂ measurements are independent of the respiration rate. VCO_2_ response only increased during exercise, even with a constant average respiration rate (RR = 20) (Figure S15, Supporting Information). Also, it was evaluated with different respiration rates while standing, and the smart‐sensing lip guard showed steady VCO_2_ responses (Figure S16, Supporting Information). The lip guard and Sentec were then compared to their CO_2_ responses during the exercise. The measured VCO_2_ from the lip guard showed a similar trend with tcPCO_2_ of the Sentec (Figure S17, Supporting Information), but it showed a delayed and smooth response because tcPCO_2_ measures diffused CO_2_ from the blood. A comparison graph of the CO_2_ response during exercise also showed matched trends between the smart‐sensing lip guard and Sentec (Figure 5C). Although Sentec is a well‐known commercial device for CO_2_ monitoring, it has been validated only for sleep monitoring. To double‐check that Sentec could represent the CO_2_ monitor during exercise, Sentec was compared with BioRadio by SpO_2_ signal. Since the subject consumes oxygen during exercise, the SpO_2_ signal showed an inverse trend against VCO_2_ (Figure S18, Supporting Information). Except for the noise signal at 3200 s, both sensors showed the same SpO_2_ trends, so Sentec can be a representative device for CO_2_ validation for exercise (Figure 5D). The osmolality of the lip guard showed an increment during exercise, as previously reported, and it was validated with a commercial MX3. The measured osmolarities of the MX3 were 60 ± 5 mOsm before exercise, 74 ± 4 during rest, and 106 ± 4 after exercise, and those results were comparable with the continuous measurements of the smart‐sensing lip guard (Figure 5E). During the incline measurement, the humidity and temperature of the environment, as well as of exhaled breath, were confirmed to be physically decoupled from each other by the 3D‐printed channel (Figure S19, Supporting Information). While the humidity of the CO_2_ sensor reached up to 81.89% during the exercise, the environmental sensor only showed 46.30% ± 3.64%, which is a negligible response compared to the CO_2_ sensor. With those results, the smart‐sensing lip guard was validated to be usable for wearable monitoring during exercise. Although the validation was assessed under the mild incline test due to the motion artifact in the commercial devices, the smart‐sensing lip guard and soft cardiac patch were qualified to be available under harsh conditions. The smart‐sensing lip guard and soft cardiac patch were evaluated during running (14.4 km h^−1^) (Figure S20, Supporting Information). VCO_2_ was measured even at a fast speed and was well matched with heart rate changes from the soft cardiac patch (Figure S21, Supporting Information).

### 2.5 Demonstration of the Integrated System Under Different Environments

**Figure 6:**
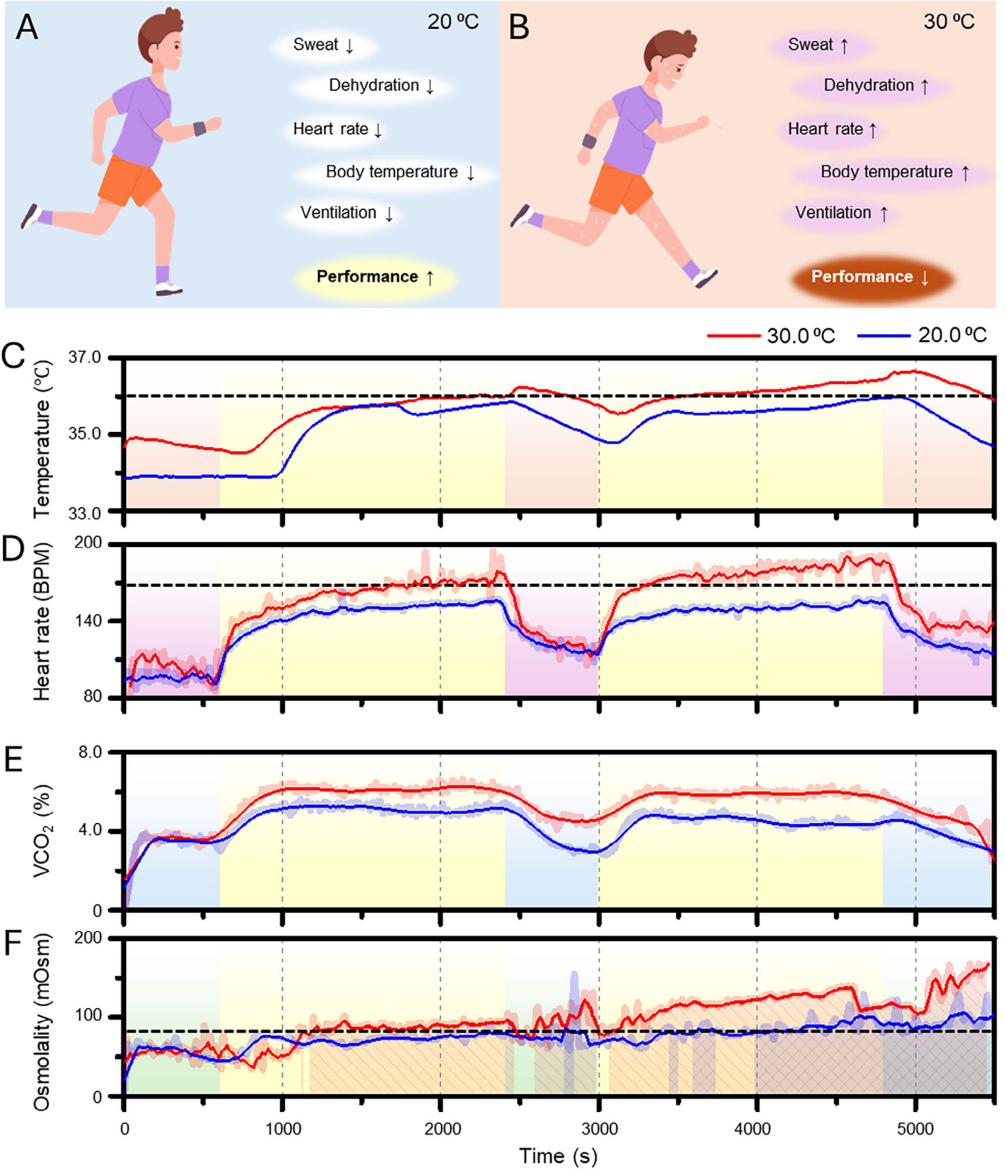
Validation of the performance of two devices under various conditions. A, B) Schematic illustration of two conditions – A) moderate temperature‐derived (20 °C) body homeostasis metabolism and B) high temperature‐derived (30 °C) homeostasis changes. C) Measured temperature changes from the soft cardiac patch during exercise under different temperature conditions. D) Measured heart rate values from the soft cardiac patch during exercise. E, F) Measured VCO_2_ values (E) and osmolality values (F) from the smart‐sensing lip guard during exercise.

The wearable soft cardiac patch and smart‐sensing lip guard were applied for exercise monitoring under different environmental conditions. It is important to monitor athletes' physiological signals for their healthcare and performance.^[^ ^28^, ^31^, ^102^ ^]^ However, most athletes' healthcare devices measure individuals' physiological data, even if it is necessary to monitor environmental factors for accurate monitoring of their performance and health because it has been reported that they can be affected. **Figure** **6**A,B describe the effect of environmental temperature on physiological signals. Under a moderate temperature (20 °C), a subject produces less sweat to maintain homeostasis and remains hydrated due to reduced moisture loss (Figure 6A).^[^ ^103^ ^]^ This condition supports a stable heart rate, body temperature, and ventilation, which makes the subject perform in the best condition. However, when the temperature is higher (30 °C), it interferes with homeostasis, and body temperature increases (Figure 6B).^[^ ^27^, ^104^ ^]^ That makes the body release a lot of sweat, which results in moisture loss.^[^ ^105^, ^106^ ^]^ To regulate the rising temperature, the heart rate increases as more blood is pumped to the capillaries. This causes the ventilation rate to increase because of the increased heart rate. Consequently, the ventilation rate also rises due to the elevated heart rate. These physiological responses ultimately reduce the subject's performance, preventing them from achieving their optimal condition.^[^ ^26^, ^107^ ^]^ The developed smart‐sensing lip guard and soft cardiac patch can measure not only physiological signals, including ECG, heart rate, osmolality, and VCO_2_, but also ambient pressure, temperature, and humidity. It is the first wearable platform to monitor the majority of physiological factors during exercise, including environmental monitoring. To compare the temperature effect, a subject exercises on the treadmill under controlled temperature conditions, 20 and 30 °C. The subject was inclined at a 15° angle and 4.8 km h^−1^ for 60 min with 10 min of rest. As shown in Figure S22 (Supporting Information), the exercise speed and strength performances were not different under varying temperature conditions. To investigate the environmental temperature effect, the humidity and atmosphere were maintained in similar conditions (Figure S23 and Video S1, Supplementary Video1). To quantify the heat stress effect in those conditions, the estimated WGBT was calculated from environmental factors using the following equation,^[^ ^108^ ^]^

(1) $$WBGT_{est}=0.567\cdot T+0.393\cdot e+3.94$$

where *T* is the temperature and *e* is the vapor pressure. The vapor pressure could be calculated by the following equation,

(2) $$e=RH\%\times 6.105\cdot \exp\left(\frac{17.27\cdot T}{237.7+T}\right)$$

where *RH*% is humidity. The estimated WGBT showed a significant difference between the two conditions, indicating that they experience different heat stress during exercise (Figure S24, Supporting Information). As a result, although the subject conducted almost the same exercise strength, the physiological signals were quite different between the two temperature conditions. The smart‐sensing lip guard and soft cardiac patch showed different physiological results under varying conditions. Figure 6C shows the skin temperature response of the soft cardiac patch under different environmental conditions (20 and 30 °C). At rest, the baseline skin temperature was ≈34.0 °C under the cooler condition (20 °C), and ≈35.0 °C under the hotter condition (30 °C), which was influenced by environmental temperature. During exercise, skin temperatures increased under both conditions due to the metabolic heat generation.^[^ ^3^, ^8^ ^]^ Under the cooler ambient conditions, the body was able to dissipate the metabolic‐generated heat effectively, with the skin temperature dropping from 35.84  to 34.78 °C during rest (ΔT = 1.06 °C), and from 35.99 to 34.73 °C during recovery (ΔT = 1.26 °C). However, under the hot temperature environment, heat dissipation was limited. In the 30 °C condition, skin temperature rose to 36.22 °C during exercise and only decreased to 35.54 °C during the recovery phase (ΔT = 0.68 °C). Similarly, during the rest phase, temperature decreased from 36.62 to 36.62 °C (ΔT = 0.68 °C), indicating inefficient heat loss. These results highlight the significant influence of environmental conditions on heat regulation. Efficient body heat release is critical for maintaining homeostasis during and after physical activity, and the observed differences emphasize the risk of heat stress in hot temperature environments.^[^ ^28^, ^30^ ^]^ The heart rate data also showed similar trends with the temperature because both physiological signals are related to heat regulation (Figure 6D). Under cooling temperature, the subject's heart rate was stable during exercise with a maximum value of 155 bpm. Heart rate recovered to 116 bpm during rest. Heart rate changes during exercise can be quantified with the target heart rate (THR) zone method.^[^ ^109^, ^110^ ^]^ The maximum heart rate can be calculated using a simple equation based on the age of a subject, as follows.

(3) $$HR_{\max}=220-Age$$

Based on the targeting zone method, exercise under cooling temperature reached the anaerobic zone with ≈80% intensity. However, under hot conditions, the heart rate quickly increased to 140 bpm and went up to a maximum of 178 bpm during the first exercise, and it was placed in the maximum zone of the THR with 95% intensity of the maximum heart rate. It was considered an extreme condition for exercise, which caused strain on the body. We set thresholds for the temperature at 36.2 °C, which is a 1.5 °C increment from the normal steady state at the cooling temperature, and heart rate at 168 bpm, which is the limited heart rate for the anaerobic zone (90% intensity). Interestingly, both physiological signals showed similar trends for exercise, even under different ambient temperature conditions. The smart‐sensing lip guard can also monitor the effect of ambient temperature because of homeostasis. An increase in heart rate led to circulation in the lungs and increased the concentration of the VCO_2_. Released VCO_2_ under hot conditions was increased by ≈1%–2% compared to cooling temperatures (Figure 6E). Frequent breath circulation also causes an increase in humidity in the exhalation of the breath. As a result, a subject loses its moisture due to sweat and breath. Figure 6F represents the osmolality of saliva. The osmolality under cooling temperature was not much increased, and it reached the dehydration range (Threshold = 80 mOsm) at ≈4000 s,^[^ ^111^ ^]^ which is the end point of the exercise. However, hot conditions made the subject dehydrated at 1200 s, and it severely increased at the endpoint. Smart‐sensing lip guard and soft cardiac patch monitor athletes' physiological signals from the face and chest, helping to assess athletic performance under varying environmental conditions. The smart‐sensing lip guard and soft cardiac patch also compared with a commercial exercise ventilation monitor Video S2, Supplementary Video2); although the commercial device offered only four signals even though it was 1.52 kg, the smart‐sensing lip guard and soft cardiac patch could offer more than eight feasible signals with high wireless connectivity and negligible weight loading (0.05 kg). Also, the participants were not obstructed by their breath due to the open channel design and lightweight features. As a result, The wearable soft cardiac patch and smart‐sensing lip guard showed simultaneous and continuous monitoring of signals, not only indicators (environmental pressure, humidity, temperature, IMU signals, and body temperature) and ECG, but also human metabolites such as VCO_2_ and salivary osmolality with a high form factor (Table S2, Supporting Information), introducing the potential of the wearable exercise monitoring device.

## 3 Conclusion 

This article introduces an all‐in‐one wireless bioelectronic system designed for athletes, capable of monitoring physiological signals, metabolites, and environmental factors during exercise. The smart‐sensing lip guard comprises an admittance osmolality sensor, a CO_2_ sensor, and an environmental humidity sensor to track facial physiological signals and surrounding conditions. Meanwhile, the soft cardiac patch measures ECG, heart rate, temperature, acceleration, and gyroscopic data, which are essential health parameters during physical activity. Experimental results with human subjects demonstrate that the device effectively detects clinical‐grade physiological signals, even when subjected to significant motion, thereby overcoming motion‐artifact issues commonly associated with conventional wearable devices. The combination of the wearable soft cardiac patch and the smart‐sensing lip guard accurately captures temperature‐dependent changes in VCO₂, heart rate, and salivary osmolality under realistic exercise conditions. Each signal related to human homeostasis, such as thermoregulation, cardiovascular strain, ventilation, and dehydration, is influenced by environmental conditions. By identifying dehydration and correlating it with heart rate and VCO₂ under various circumstances, new insights into exercise performance monitoring in relation to environmental factors are gained. Collectively, the class of technologies developed in this work lays a strong foundation for future advancements in personalized performance optimization, heat stress management, and athletic healthcare applications. Moreover, the demonstrated multimodal integration of environmental and metabolic signals enables early detection of exercise‐induced abnormalities through comprehensive studies, providing a scalable framework for broader applications in sports healthcare.

## 4 Experimental Section

### Materials

Copper foil (6 µm, MSE Supplies), polyimide tape (Kapton tape, Bertech), polyimide film (Kapton film, Dupont), polydimethylsiloxane (PDMS), Chloroauric acid (HAuCl_4_, Sigma Aldrich), solder paste (Chip Quik), tacky flux (Chip Quik) were purchased for manufacturing and soldering the wearable soft cardiac patch and smart‐sensing lip guard.

### Instruments

A femtosecond infrared laser micromachine (OPTEC) was used for the microfabrication of the electrodes. A force measurement instrument (Mark‐10) was used to measure the long‐term stability of the electrodes. An e‐beam evaporator (Denton Explorer) was used to deposit gold (Au). A hot press (Rositek) was used for thermal attachment between polyimide films. A 3D printer (ELEGOO; Saturn 4) was used for manufacturing the tidal gas channel. A potentiostat (Gamry, Interface 1010B) was used for impedance measurement.

### Preparation of Capacitive Osmolality Sensor

A clean aluminum plate was spin‐coated with PDMS (Sylgard 184, Dow) using a ratio of 1:5 for the curing agent to base mixture. Then, the copper foil was laminated onto the PDMS‐coated aluminum plate and cleaned with isopropanol. The extra copper foil was removed from the plate, and a heat‐responsive polyimide (PI) sheet was covered on the copper with a heat press at 350 °C for 20 min. Copper/PI was then patterned with a 100‐micrometer gap from the electrode, and its cover was prepared with PI using femtosecond laser micromachining (Optec laser systems, Belgium). To complete the encapsulation process of the copper electrode, the cover was placed on the exposed surface of the copper electrode, and a heat press was applied at 350 °C for 20 min. The electrode was reductively deposited with gold from AuHCl_4_ solution with a power of 3 V and 0.1 mA for 30 s. Laminated copper wires with a diameter of 0.3 mm were connected to the capacitive copper sensor, finalizing the assembly. For long‐term stability, the sensor was dipped and continuously measured its admittance every hour.

### Preparation of ECG Gold Serpentine Electrodes

For an ECG gold serpentine electrode, PDMS was spin‐coated on the glass slide. The PI film was then covered on the glass slide, and Au was deposited. To make a stable PI/Au layer, 10 nm of Cr was deposited by e‐beam evaporation under 1 Å s^−1^, and 200 nm of Au was then deposited with a condition of 5 Å s^−1^. After the ultrathin Au plate was fabricated on the slide, it was laminated with a femtosecond IR laser for the serpentine pattern, and excess Au/PI film was peeled off. They are transferred to the water‐soluble tape before being transferred to the medical tape. For long‐term cyclability, resistance was continuously measured with 90° twisting and 10% stretching repeatability with Mark‐10.

### Wearable Smart‐Sensing Lip Guard and Soft Cardiac Patch Real‐Time Data Acquisition

The wearable smart‐sensing lip guard and soft cardiac patch were designed with Altium for a compact fPCB layout, placing components on both sides. Soldering was performed in the laboratory with conductive solder paint and flux at 180 °C. Integrating various sensors is key to the devices' functionality. The smart‐sensing lip guard integrates a low‐power system through analog‐to‐digital converters (ADC) to convert analog signals from sensors into digital information, which is then processed by the NRF52832 microcontroller unit (MCU). The AD5933 is utilized as a precision impedance converter for osmolality measurements. To determine the frequency range of the AD5933, the wearable smart‐sensing lip guard was measured for impedance using a potentiostat, with a range of 100 to 100 Hz. The AD5933 impedance converter was then configured to sweep frequencies from 10 to 15 kHz in 100 Hz increments (51 points in total). This linear‐response mid‐frequency range was selected to minimize electrode polarization effect and non‐ideal capacitive behavior observed at lower frequencies (<10 kHz) and to avoid parasitic capacitive effects in flexible substrates that become significant at higher frequencies (>100 kHz). The chosen window stabilizes the admittance response for salivary electrolyte sensing, avoiding low‐frequency artifacts under dynamic, bio‐integrated conditions. SCD‐41 and BME280 sensors provide accurate CO_2_ and humidity readings. Operating at 3.3 V, the smart‐sensing lip guard achieves efficient power utilization, consuming 16.5 mW during active sampling. This optimized power design extends battery life to ≈14 h using a 70‐mAh LiPo battery, balancing energy efficiency with high sampling rates for reliable performance. Similarly, the soft cardiac patch incorporates a system with ADCs to digitize analog signals, which are also processed by the NRF52832 MCU. This device integrates the ADS1292 channel converter for ECG measurements at 250 Hz and the ICM20948 and TMP117 sensors for inertial measurement units (IMUs) and temperature monitoring, respectively. The soft cardiac patch is optimized for real‐time, accurate data collection and operates with updated low‐power technical specifications, including a 3.3 V operating voltage, 23.1 mW total power consumption, and a battery life of ≈10 h with a 70‐mAh LiPo battery. Both system‐embedded built‐in active shield drivers effectively manage electromagnetic interference (EMI) for precise high‐sampling readings. Integrating these multimodal architectures enables the smart‐sensing lip guard to provide precise, real‐time data acquisition for athlete monitoring.

### Integration of the Smart‐Sensing Lip Guard

A commercial lip guard (Shock Doctor, USA) featuring an interchangeable mouthpiece and lip guard was modified to incorporate the admittance sensing circuit and a Li‐Po battery. The lip guard surface was cleaned using acetone and isopropanol to remove any existing coating layers. Then, epoxy (Loctite) was applied to the treated surface to secure the circuitry and battery. fPCB was bent to fit the curvature of the lip guard so it helps seamless design. For breath trapping on the lip guard, a 3D‐printed channel was designed. SCD‐41 CO_2_ sensor was placed at the center of the lip guard, and it was covered with the channel and fixed with epoxy. For breath simulation, COMSOL was used for an exhale‐inhale model with the 3D printed channel based on the validated mask model.

### Assembly of the Soft Cardiac Patch

To apply the soft cardiac patch device on the human skin, 3 M medical tape (3M‐4076) was used as an adhesive layer. The medical tape was first laminated with laser for the patch design, and the surface, except for the area of the circuit and electrodes, was punched with a 1 mm radius to help with sweat removal. The electrodes on the water‐soluble tape were transferred to the medical tape, and they were cleaned with deionized water. fPCB was placed on the center of the medical tape, with an ecoflex silicone damping layer (5 mm). To connect between the electrodes and the fPCB, 40 µm of copper wire was used. All of the electric regions except for the electrodes were covered by ecoflex.

### Validation of the Wearable Smart‐Sensing Lip Guard and Soft Cardiac Patch with Commercial Devices

The human subject study was conducted with the participation of several healthy volunteers, adhering to the IRB‐approved protocol (#H23170) from the Georgia Institute of Technology. The signals from the wearable soft cardiac patch were compared with the validated commercial devices. The subject wore the soft cardiac patch, heart rate meter (Polar HR), and motion sensor (LSM6DSV), and they were compared during running. To avoid interference between devices, the soft cardiac patch was placed on the center of the sternum, the heart rate meter was placed on the xiphisternum, and the motion sensor was placed on the back with a strap. The subject did exercise with different velocities (4.3, 5.4, 10.8, and 14.4 km h^−1^). The soft cardiac patch measures heart rate from its ECG signal, and it was compared with the heart rate meter. The total acceleration was calculated from 3‐plane IMU signals, and it was compared with the motion sensor. The velocity was calculated from the total acceleration data, with integration over time. To validate the smart‐sensing lip guard, commercially available products were compared. The subject wore a lip guard, soft cardiac patch, tcPCO_2_ sensor (Sentec), and fingertip SpO_2_ device (BioRadio). Real‐time validation demonstrated the smart‐sensing lip guard's ability to measure VCO_2_ and salivary osmolality, aligning closely with tcPCO_2_ and commercial benchmarks. For the comparison of the salivary osmolality, a salivary dehydration meter (MX3) was used when the subject wore off the lip guard for 1 min before exercise, during rest, and after exercise. To ensure their reliability, the heart rate from the soft cardiac patch, Sentec, and BioRadio were compared. Also, the SpO_2_ signal from the Sentec was compared with the BioRadio to double‐check the Sentec device.

### Demonstration of the Wearable Smart‐Sensing Lip Guard and Soft Cardiac Patch with Different Temperature Conditions

The human subject study was conducted with the participation of several healthy volunteers, adhering to the IRB‐approved protocol (#H23170) from the Georgia Institute of Technology. All participants provided informed consent by signing the requisite forms, thereby authorizing the experimental procedures. Before participating in the test, subjects were required to abstain from eating and drinking (including water) after their evening meal. Upon arrival in the morning at the test location, nude body mass was initially recorded. Concurrently, the subject wore the wearable smart‐sensing lip guard on their mouth and soft cardiac patch on their sternum, and the subject exercised in the closed patient room under varying environmental temperature conditions. To keep the subject safe, all sessions were conducted with a mild exercise condition, with a 15° and 4.8 km h^−1^ speed. Each session was a 1‐h exercise with a 10‐min resting time between the exercises. After exercise, the subject kept wearing the device for 15 min to monitor the recovery session.

## Conflicts of interest

The authors declare no conflict of interest.

## Author Contributions

T.W.K., K.R.K., and Y.J.L. contributed equally to this work. T.W.K., K.R.K., Y.J.L., and W.Y. conceptualized the work. T.W.K., K.R.K., Y.J.L., H.K., S.H.L., H.Y., H.S.K., and H.K. performed the experiments. T.W.K., K.R.K., and Y.J.L. analyzed the data. Y.K. conducted a simulation analysis. T.W.K., K.R.K., Y.J.L., M.M.‐S., and W.Y. wrote a paper.

## Supporting information



Supporting Information

Supplementary Video1

Supplementary Video2

## Data Availability

The data that support the findings of this study are available from the corresponding author upon reasonable request.
