# Supplementary material for: Wireless Soft Athlete Bioelectronics for Monitoring Carbon Dioxide Ventilation and Physiological Performance
Source: Adv Sci (Weinh). 2025 Jun 20;12(34):e03880. doi: 10.1002/advs.202503880 (PMC12442608; doi:10.1002/advs.202503880)
Supplement: Supplementary file 1 — Supporting Information [file ADVS-12-e03880-s002.pdf]

ADVANCED SCIENCE
Open Access

Supporting Information

for *Adv. Sci.*, DOI 10.1002/advs.202503880

Wireless Soft Athlete Bioelectronics for Monitoring Carbon Dioxide Ventilation and Physiological Performance

*Tae Woog Kang, Ka Ram Kim, Yoon Jae Lee, Hodam Kim, Sung Hoon Lee, Youngjin Kwon, Hoon Yi, Hojoong Kim, Hyeonseok Kim, Alec Harp, Jud Ready, Melinda Millard-Stafford and Woon-Hong Yeo**

Supporting Information

Wireless Soft Athlete Bioelectronics for Monitoring Carbon Dioxide Ventilation and Physiological Performance

*Tae Woog Kang, Ka Ram Kim, Yoon Jae Lee, Hodam Kim, Sung Hoon Lee, Youngjin Kwon, Hoon Yi, Hojoong Kim, Hyeon Seok Kim, Alec Harp, Jud Ready, Melinda Millard-Stafford, and Woon-Hong Yeo**

Dr. T. W. Kang, Dr. K. R. Kim, Dr. H. Kim, Dr. H. Yi, Y. Kwon, Dr. H. Kim, Dr. H. S. Kim, and Prof. W.-H. Yeo
George W. Woodruff School of Mechanical Engineering, College of Engineering, Georgia Institute of Technology, Atlanta, GA 30332, USA
Dr. T. W. Kang, Dr. K. R. Kim, Y. J. Lee, Dr. H. Kim, Dr. S. H. Lee, Dr. H. Yi, Y. Kwon, Dr. H. Kim, Dr. H. S. Kim, and Prof. W. -H. Yeo
Wearable Intelligent Systems and Healthcare Center (WISH Center), Institute for Matter and Systems, Georgia Institute of Technology, Atlanta, GA 30332, USA
Dr. Y. J. Lee
School of Electrical and Computer Engineering, Georgia Institute of Technology, Atlanta, Georgia 30332, USA
Y. Kwon
School of Materials Science and Engineering, Georgia Institute of Technology, Atlanta, Georgia 30332, USA
A. Harp and Dr. M. Millard-Stafford
School of Biological Sciences, Georgia Institute of Technology, Atlanta, Georgia 30332, USA
Dr. Jud Ready
Electro-Optical Systems Laboratory, Georgia Tech Research Institute, Atlanta, Georgia 30332, USA
Dr. W.-H. Yeo
Parker H. Petit Institute for Bioengineering and Biosciences, Georgia Institute of Technology, Atlanta, Georgia 30332, USA
Dr. W.-H. Yeo
Korea KIAT-Georgia Tech Semiconductor Electronics Center (K-GTSEC), Institute for Matter and Systems, Georgia Institute of Technology, Atlanta, GA 30332, USA
*E-mail: whyeo@gatech.edu

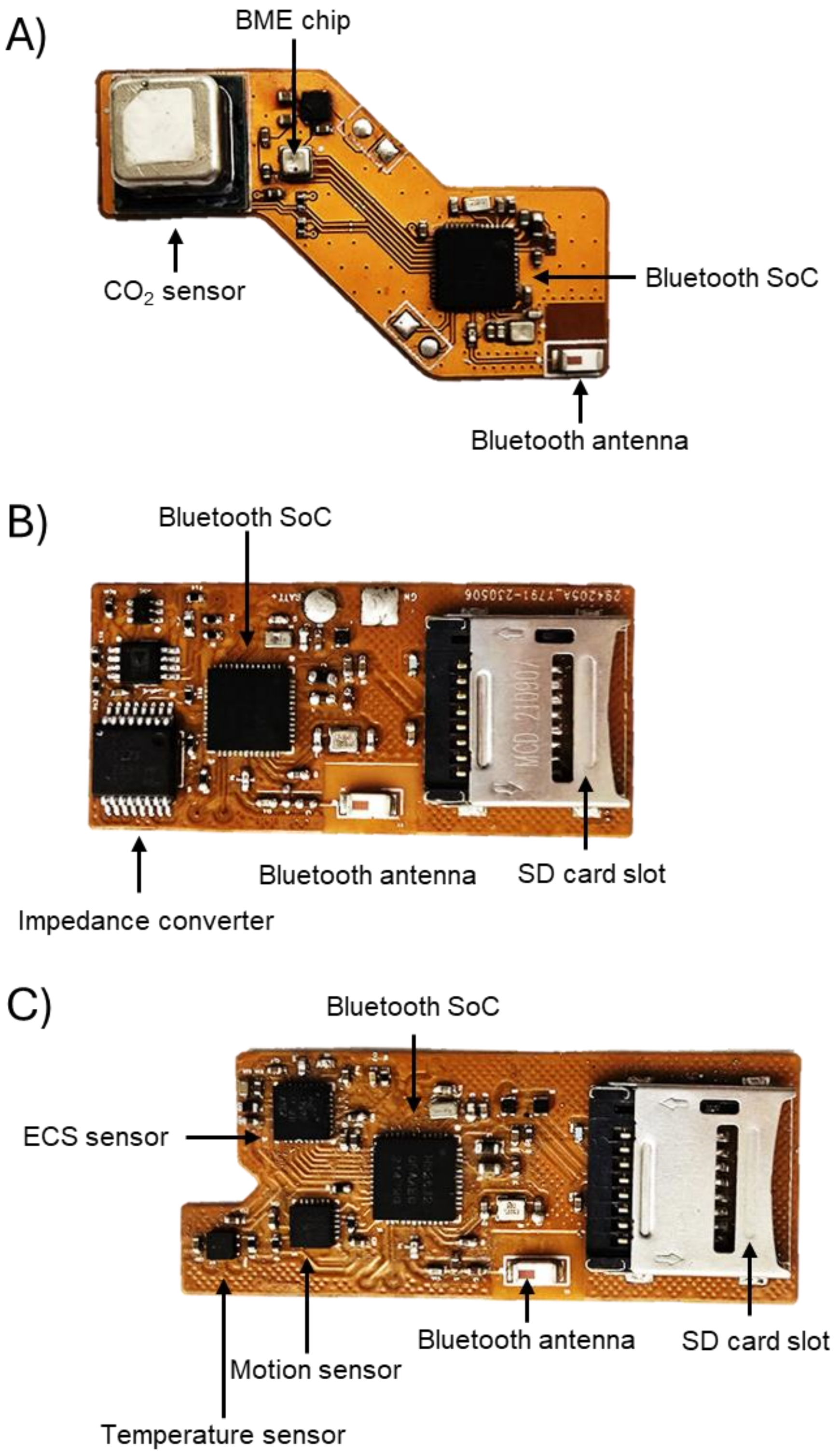

Supporting Figure S1. Flexible printed circuit boards (fPCB) for the smart-sensing lip guard and soft cardiac patch. A) fPCB for the CO_2 and ambient condition monitoring, B) fPCB for admittance osmolality sensing, and C) fPCB for the soft cardiac patch.

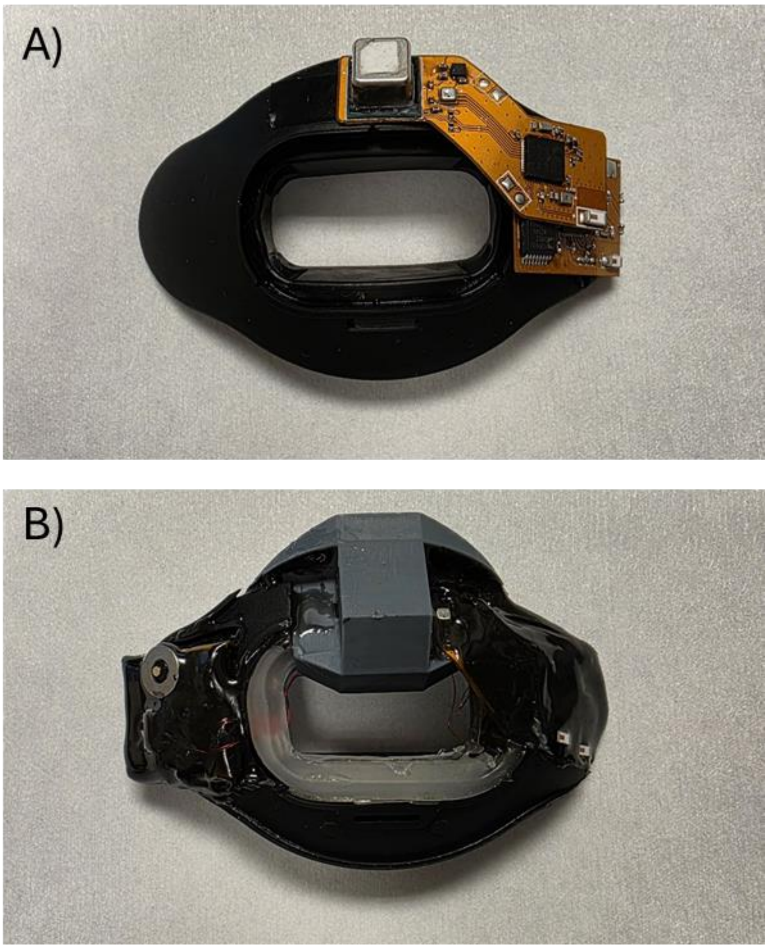

Supporting Figure S2. Front view of the smart-sensing lip guard A) before and B) after assembly.

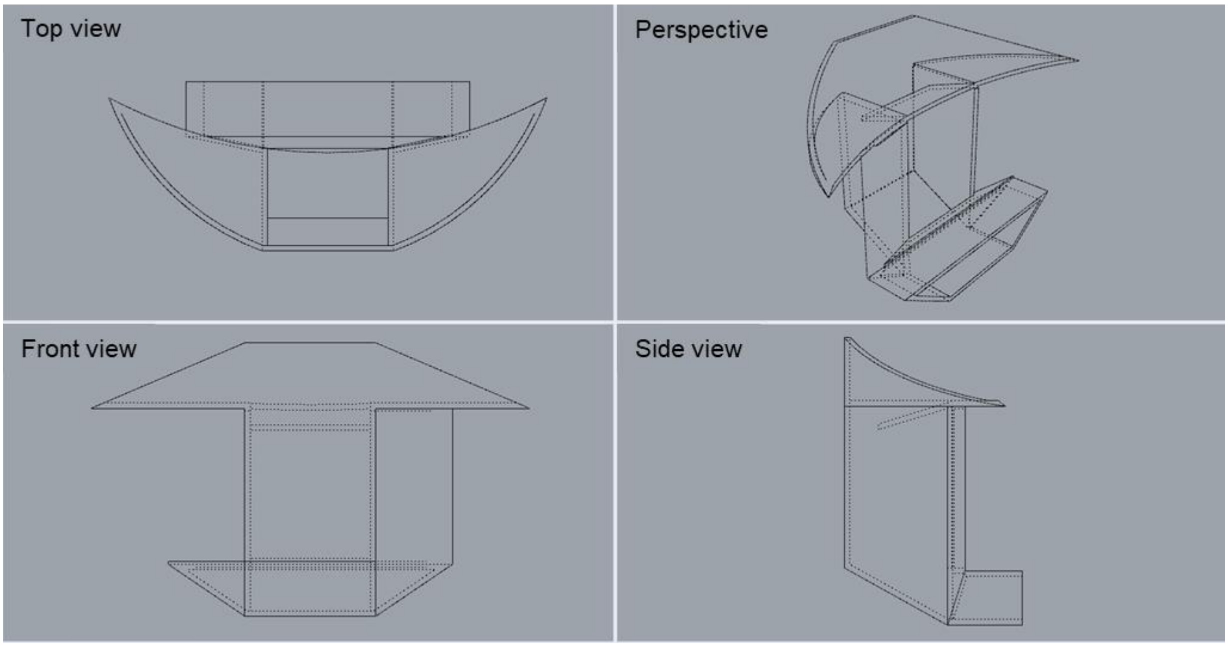

Supporting Figure S3. Planar figure of the 3D-printed breath collecting channel.

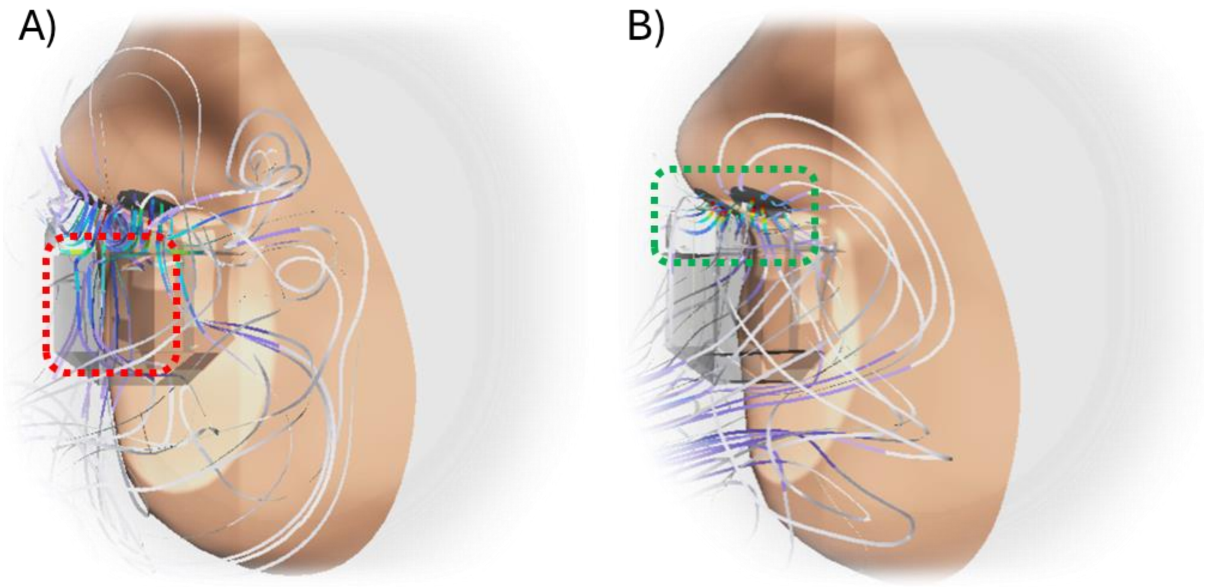

Supporting Figure S4. Simulated air flow with the 3D-printed channel with A) exhalation and B) inhalation from the nose.

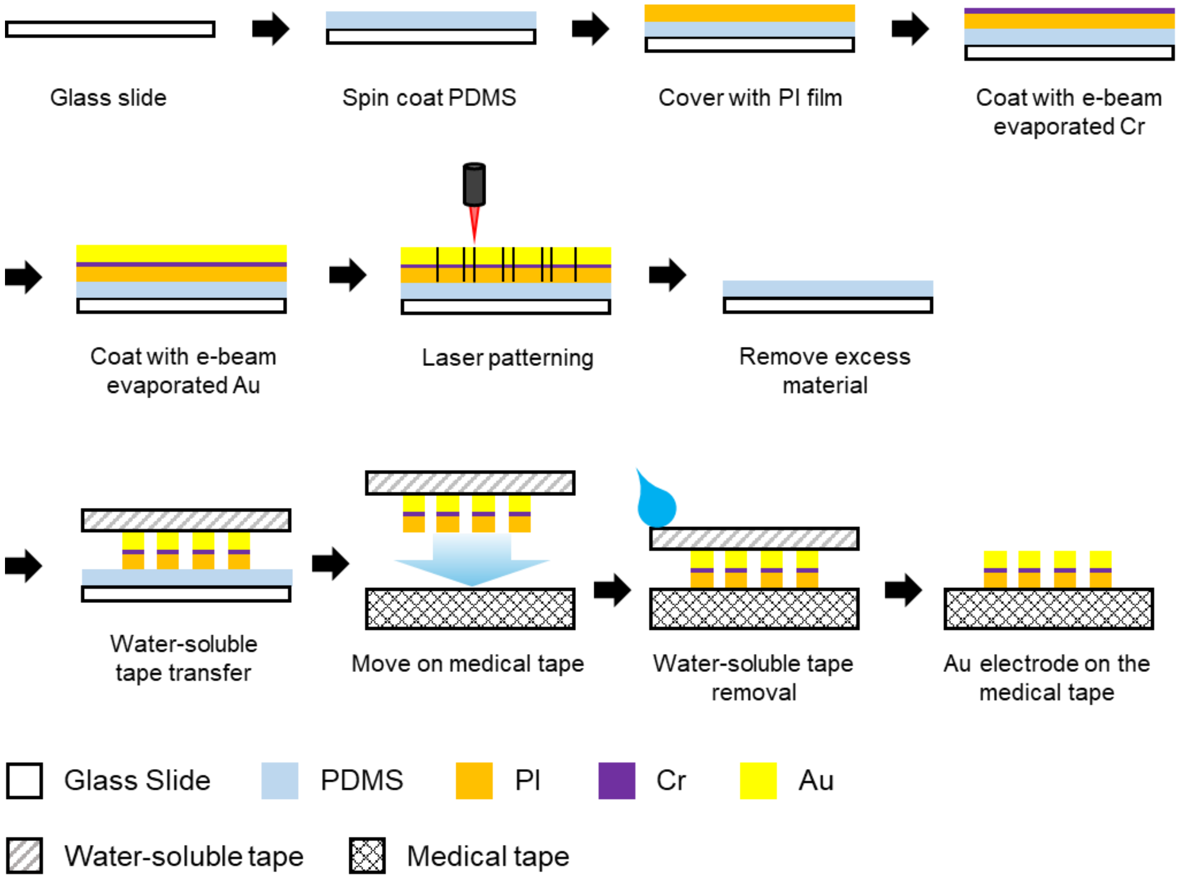

Supporting Figure S5. Synthesis of the serpentine gold electrode.

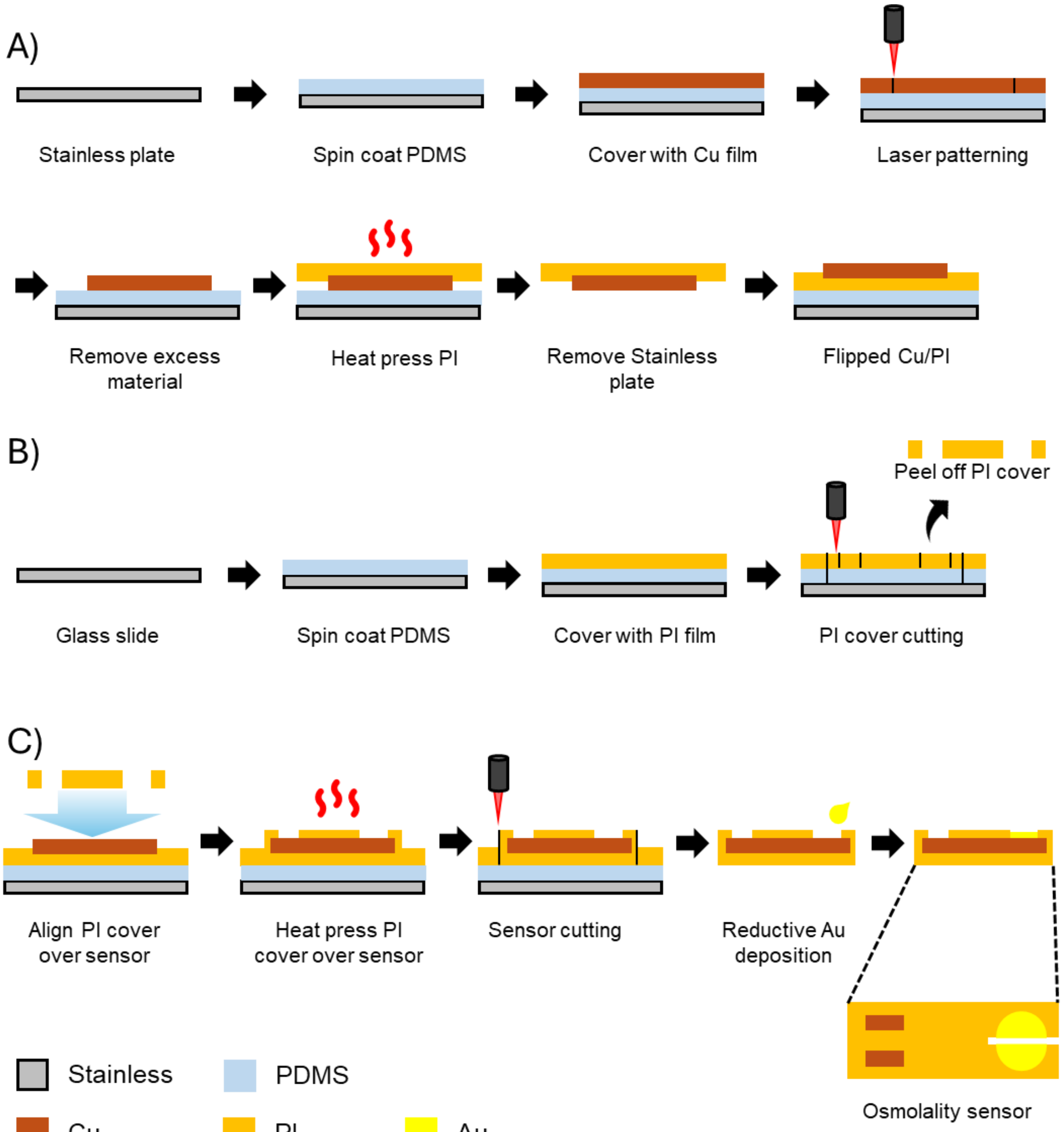

Supporting Figure S6. Synthesis of the admittance osmolality sensor. A) Preparation of the copper/PI electrode, B) fabrication of the PI sensor cover, and C) assembly of the gold-deposited osmolality sensor.

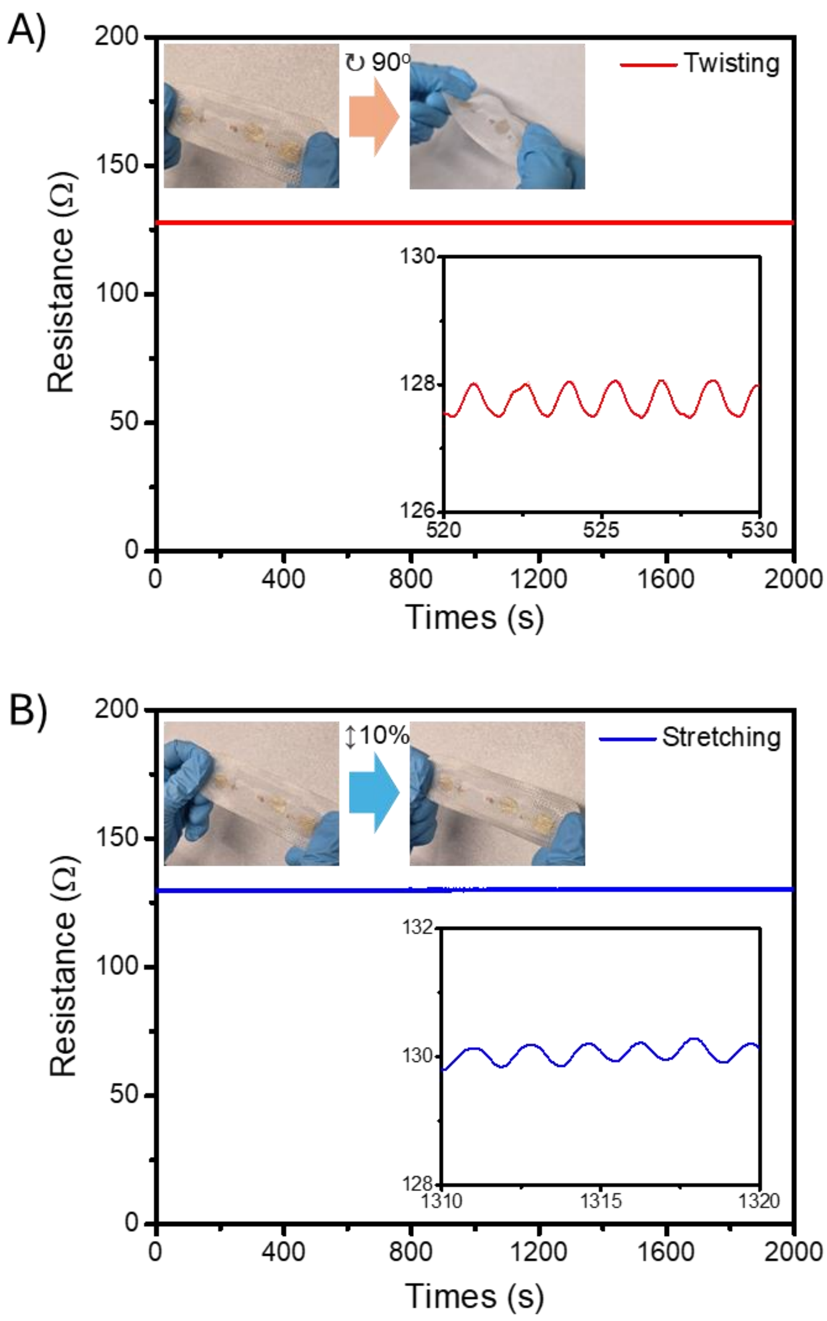

Supporting Figure S7. ECG electrode stability with repeating A) 90° twisting and B) 10% stretching for 1200 cycles.

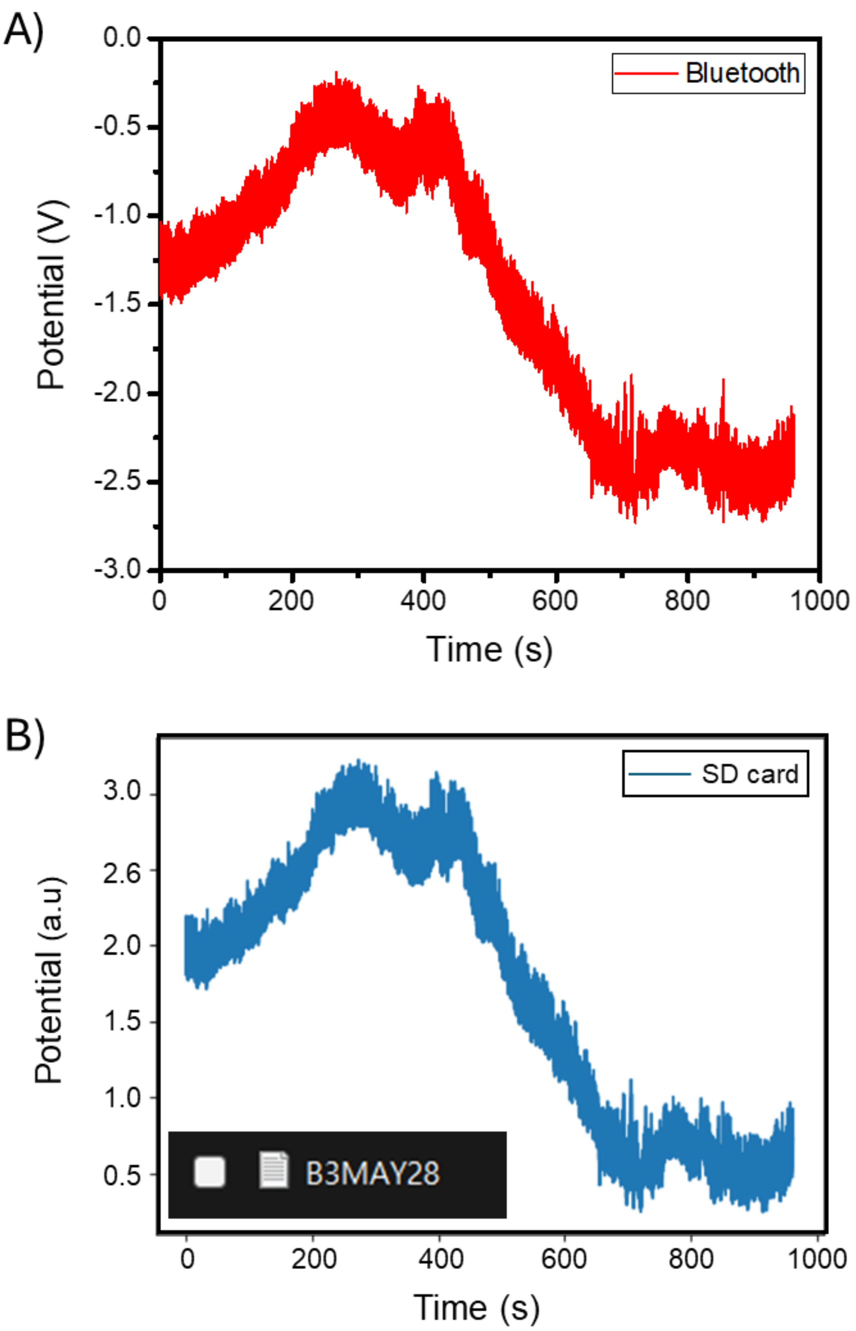

Supporting Figure S8. Raw data of the ECG electrode from A) Bluetooth communication and B) SD card stored data.

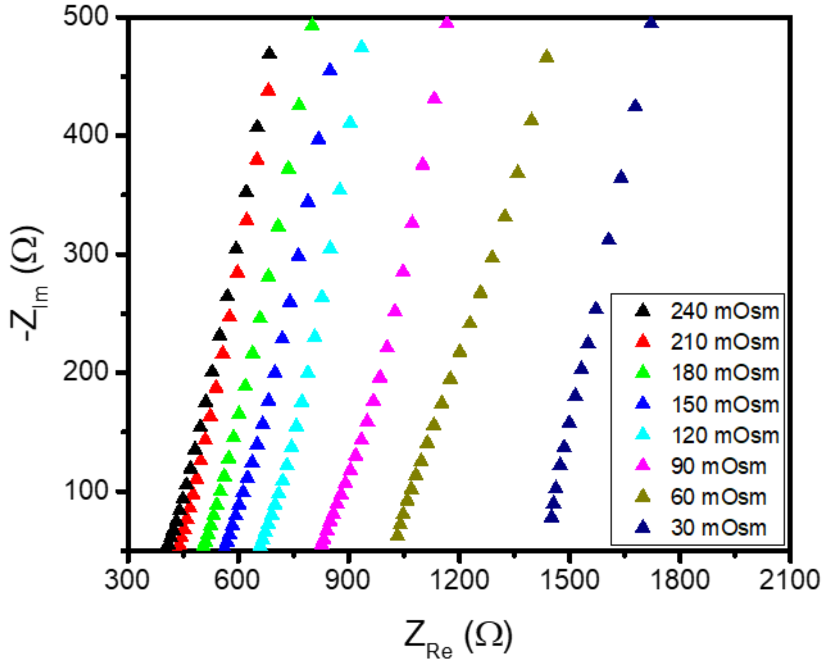

Supporting Figure S9. Nyquist plot of the admittance osmolality sensor under different concentrations of the artificial electrolyte.

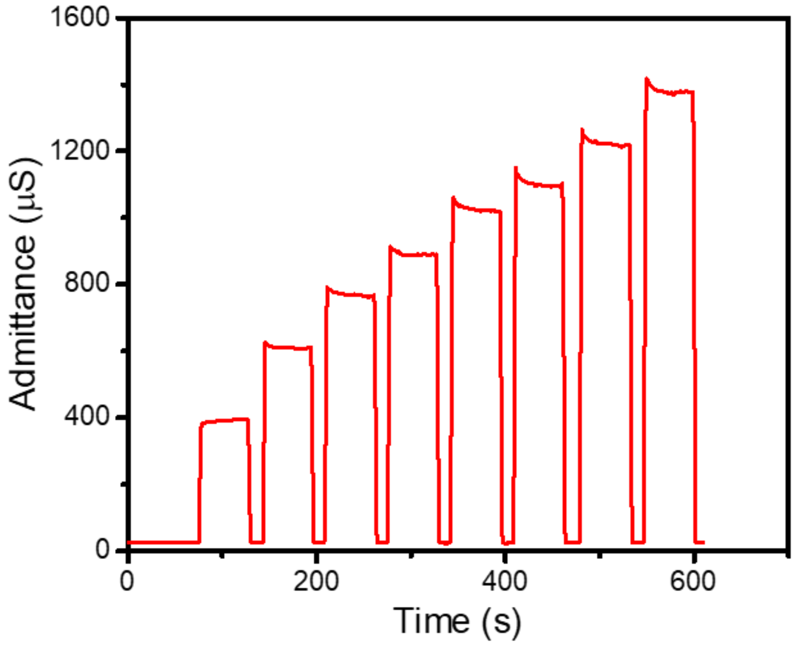

Supporting Figure S10. Admittance measurements of the osmolality sensor under varying electrolyte concentrations from 30, 60, 90, 120, 150, 180, 210, and 240 mOsm.

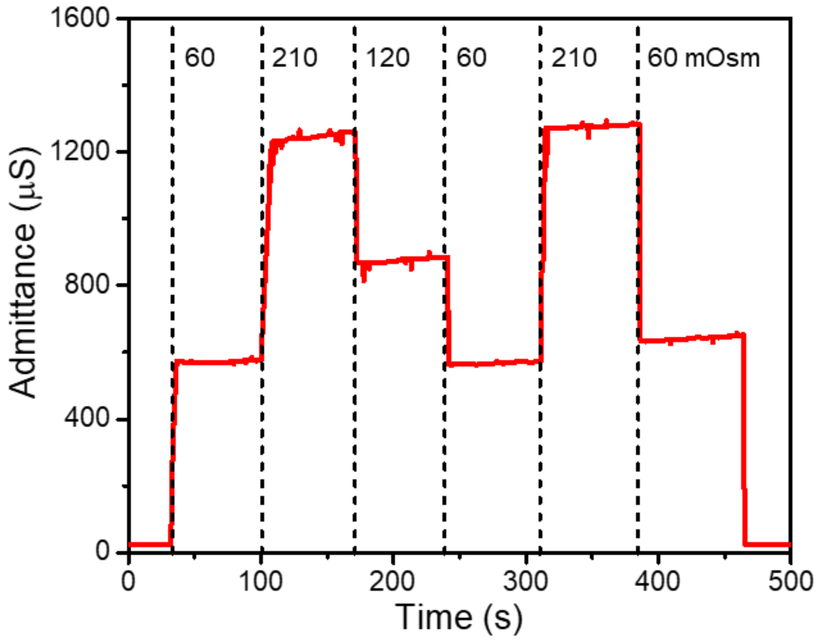

Supporting Figure S11. Continuous measurements of the osmolality sensor dipping in random electrolyte concentrations among 60, 120, and 210 mOsm.

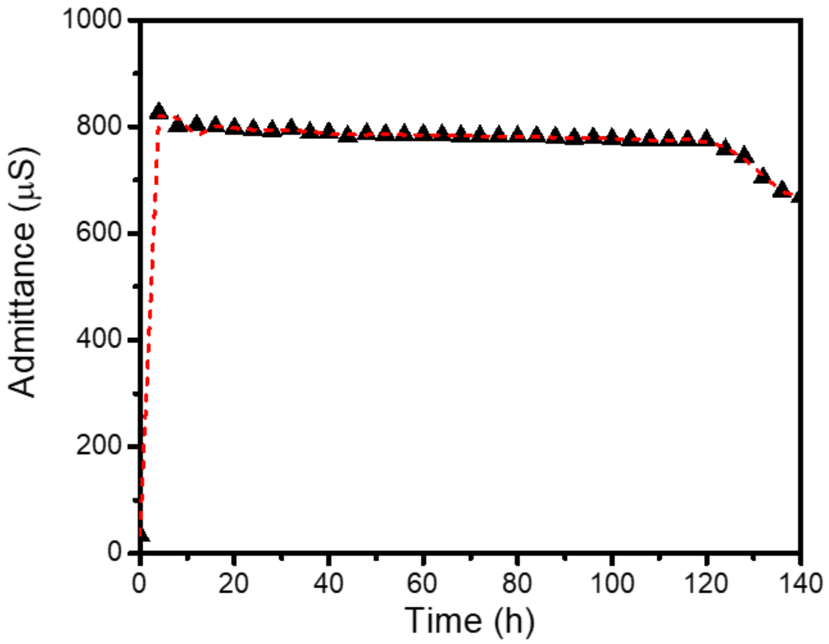

Supporting Figure S12. Long-term stability of the osmolality sensor dipping 90 mOsm.

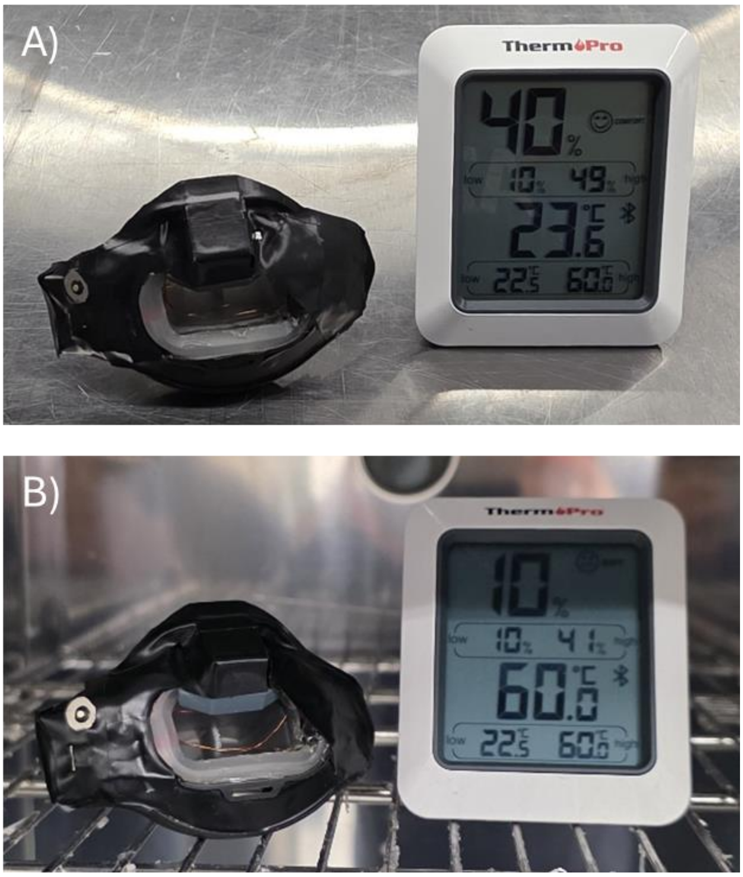

Supporting Figure S13. A) Temperature and humidity response of the commercial sensor before being put in the oven (room temperature), B) after being put in the oven.

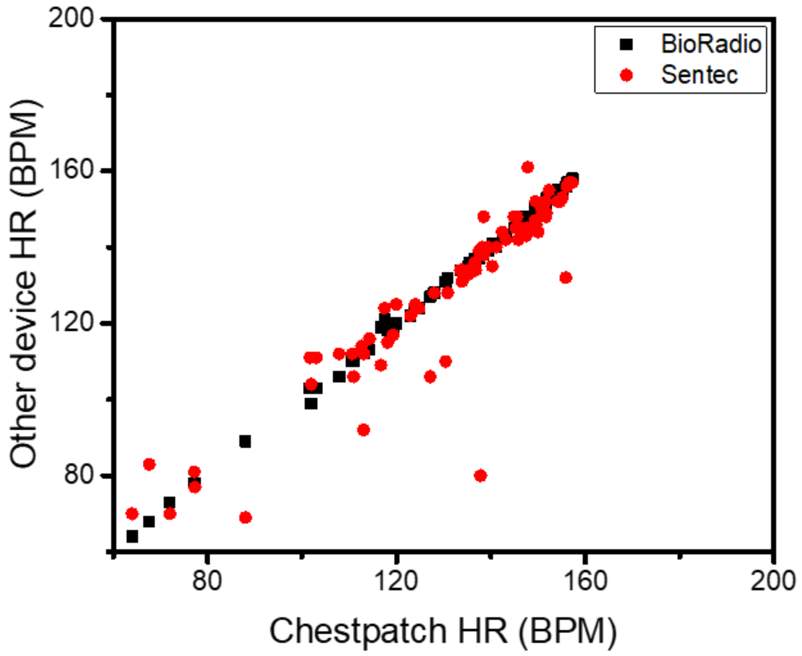

Supporting Figure S14. Comparison of the heart rate between the soft cardiac patch and the other devices.

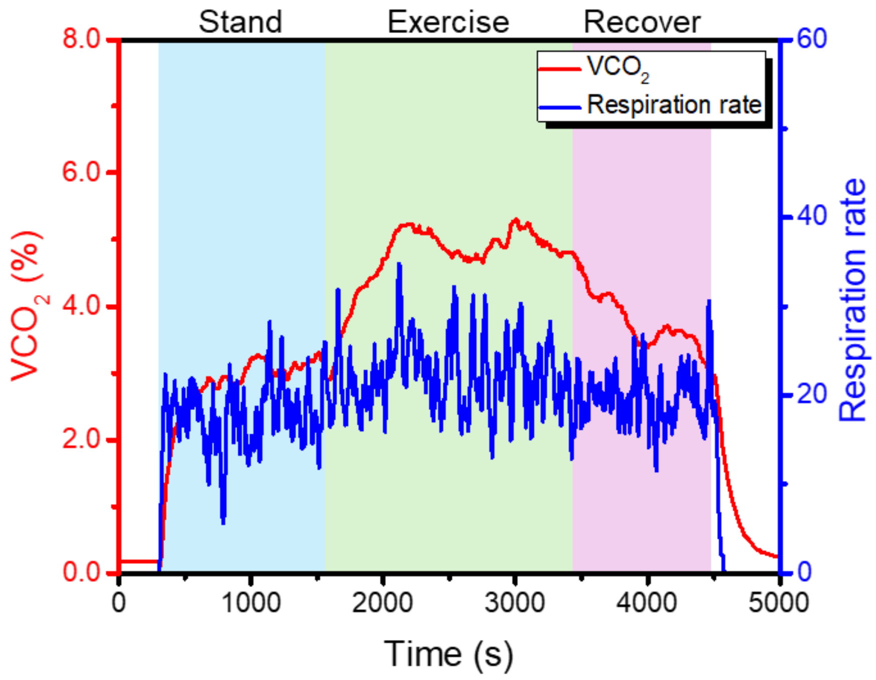

Supporting Figure S15. Comparison between VCO_2 and respiration rate during different states.

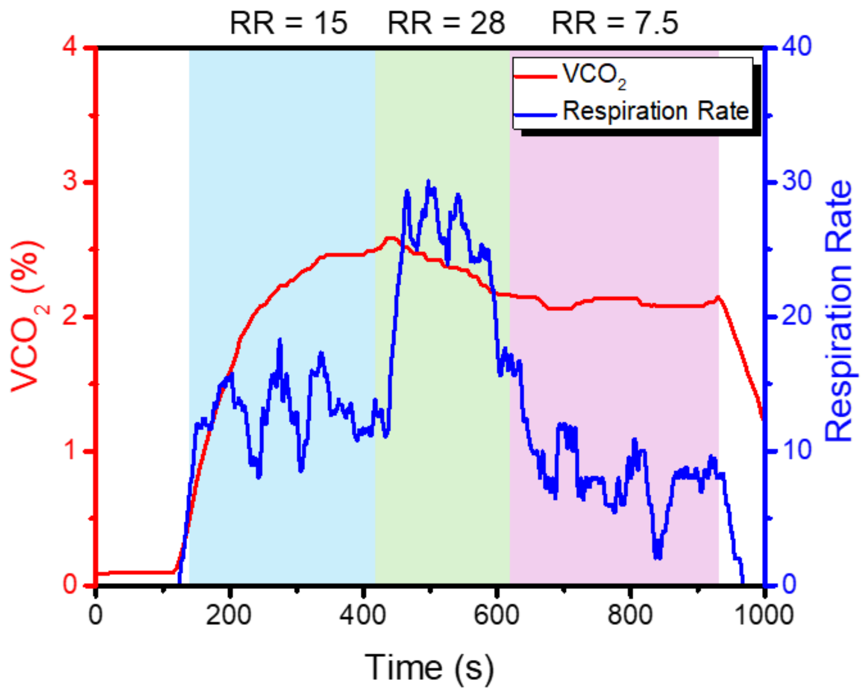

Supporting Figure S16. Comparison between VCO_2 and respiration rate with different respiration rates.

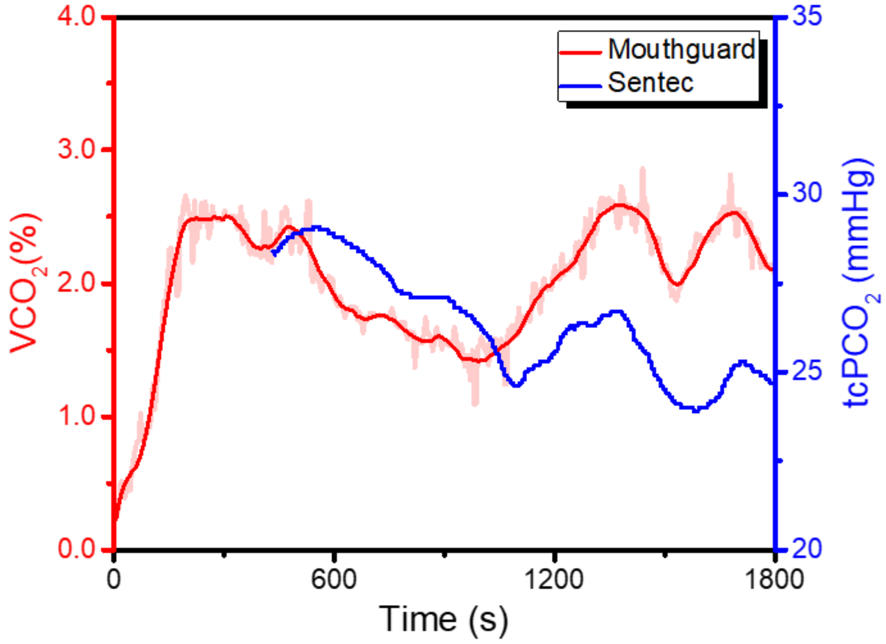

Supporting Figure S17. Comparison of the CO_2 measurement between the smart-sensing lip guard and Sentec without exercise.

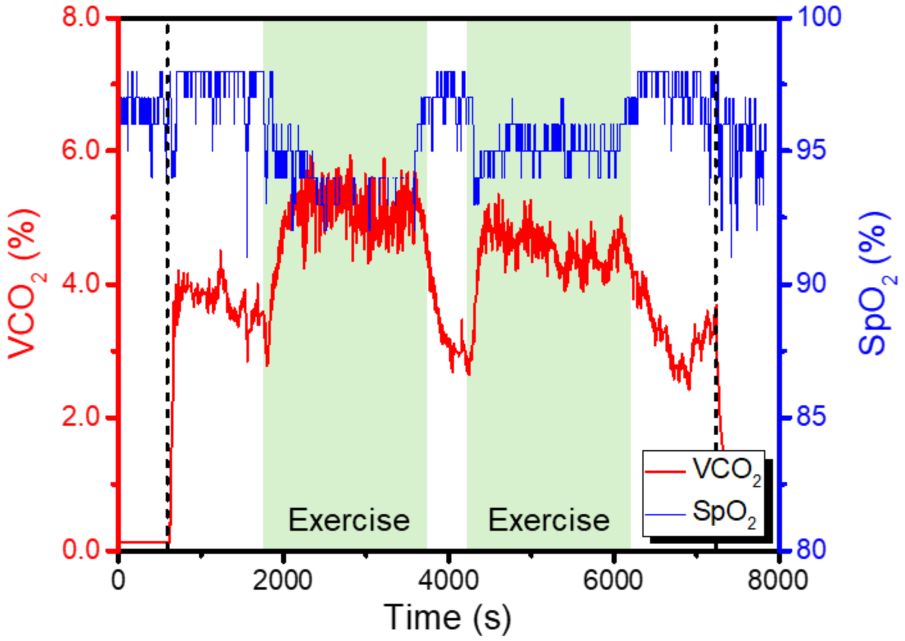

Supporting Figure S18. Comparison between VCO_2 from the smart-sensing lip guard and SpO_2 from the BioRadio.

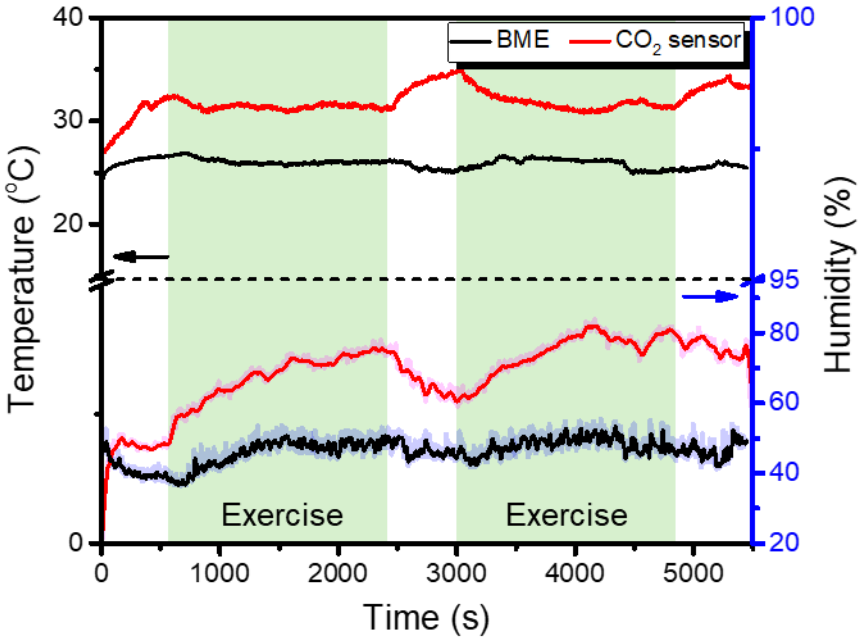

Supporting Figure S19. Measured temperature (upper) and humidity (lower) from the BME chip and CO_2 sensor of the smart-sensing lip guard during the indoor treadmill exercise.

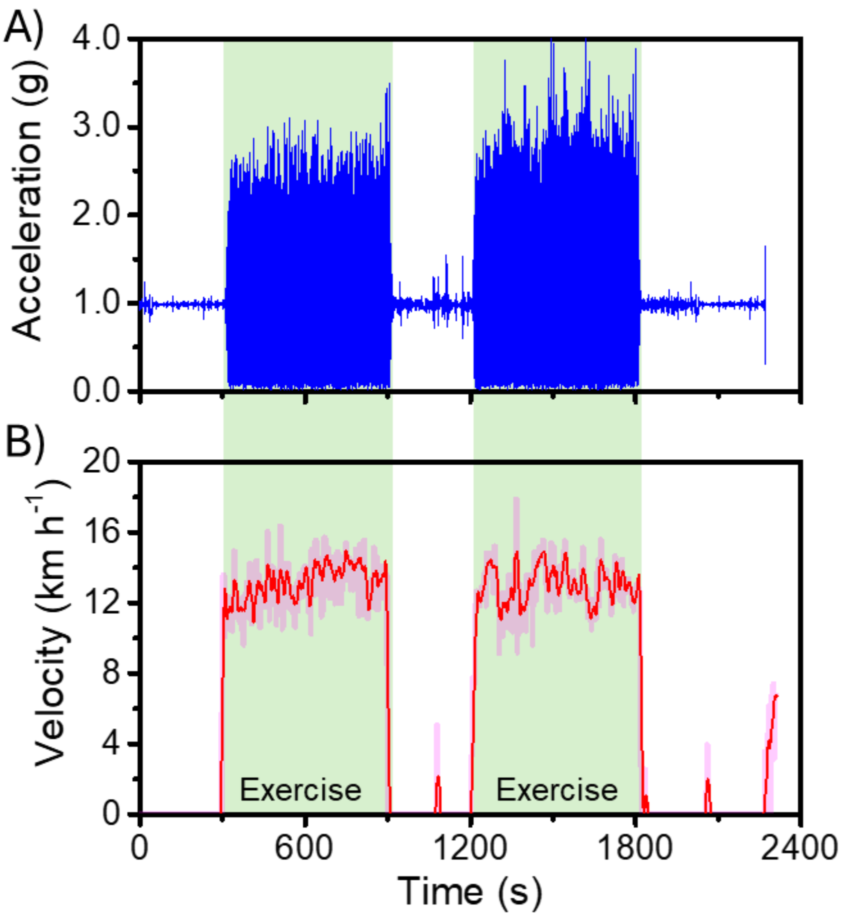

Supporting Figure S20. Measured acceleration and velocity from the soft cardiac patch during running (14 km h^{-1}).

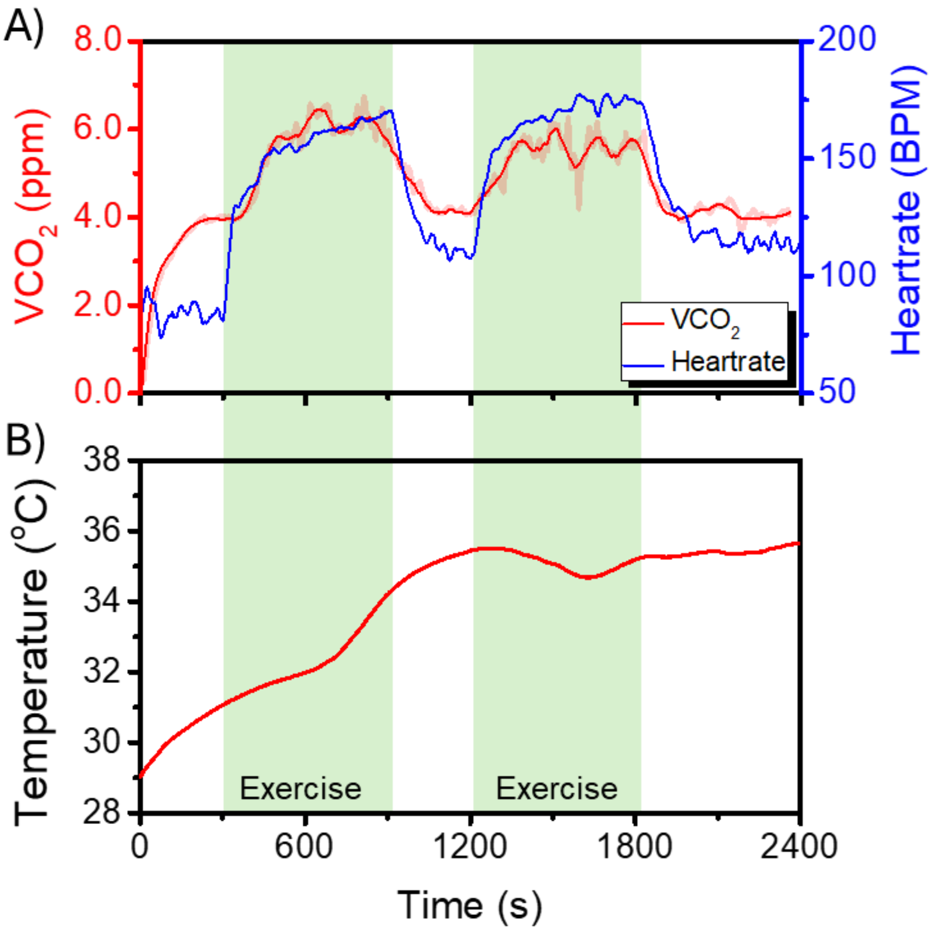

Supporting Figure S21. A) Measured VCO2, heart rate, and B) body temperature from the smart-sensing lip guard and soft cardiac patch during running (14 km h^{-1}).

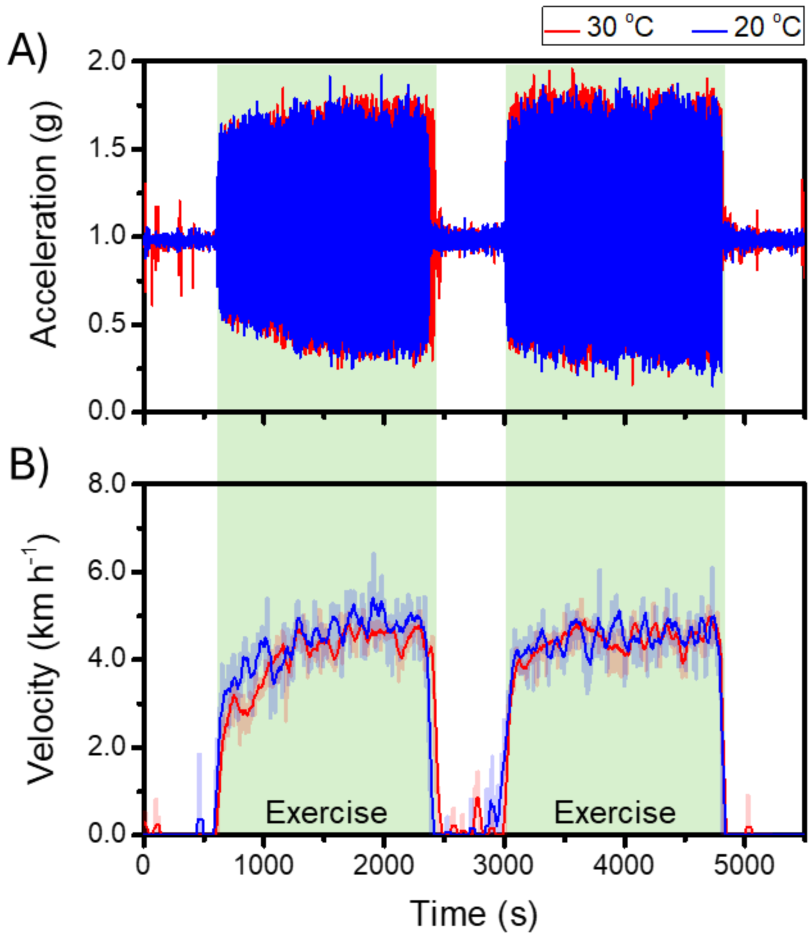

Supporting Figure S22. Acceleration and velocity comparison under different temperature controls.

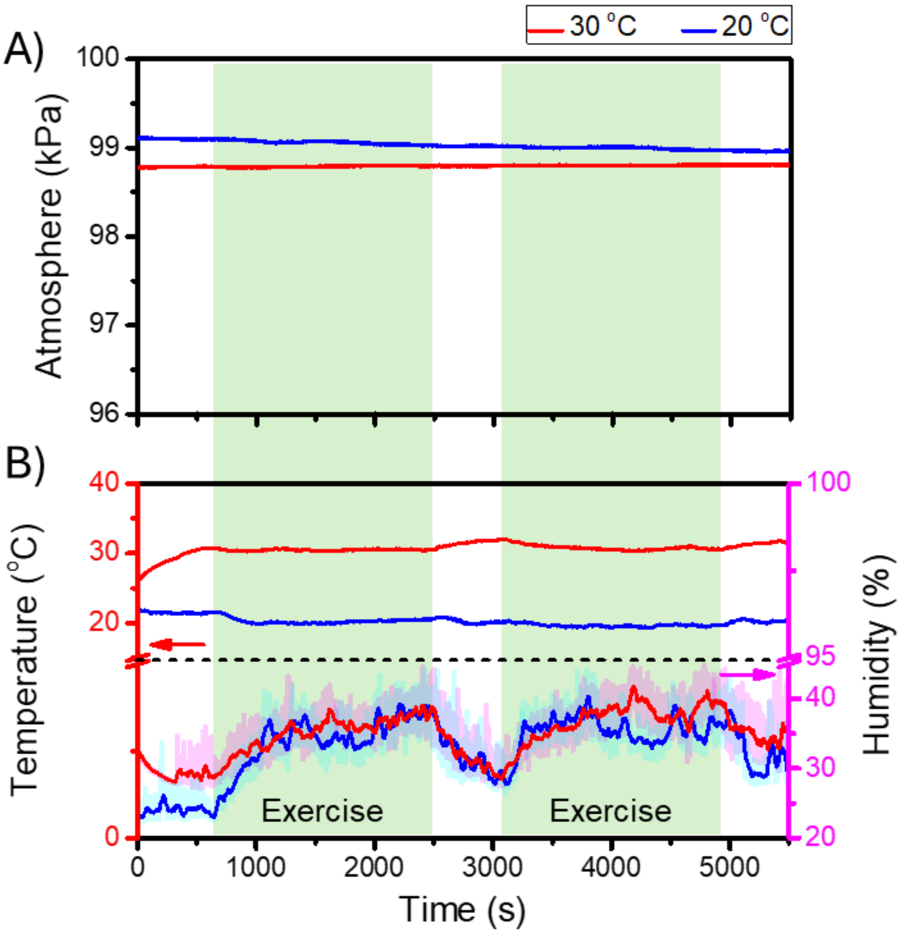

Supporting Figure S23. A) Measured ambient atmospheric pressure and B) temperature (upper) and humidity (lower) from the smart-sensing lip guard under different temperature controls.

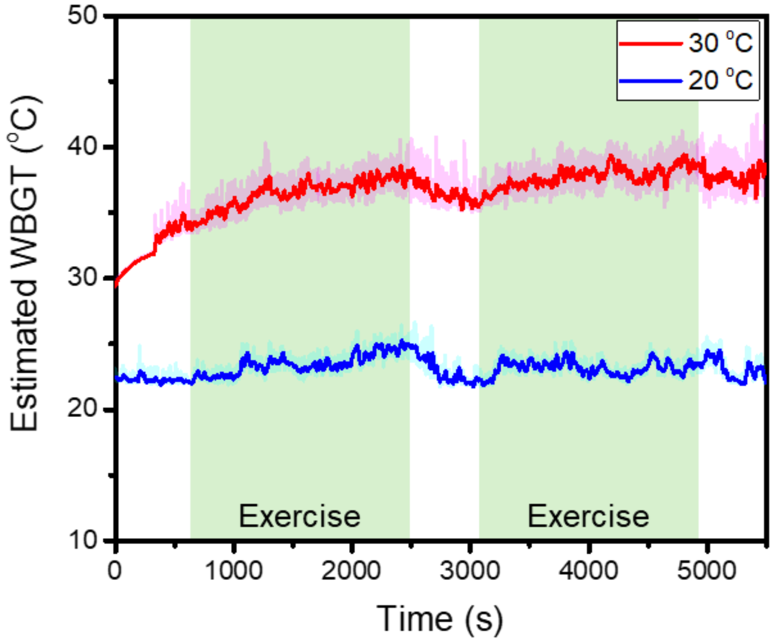

Supporting Figure S24. Estimated Wet-Bulb Globe Temperature (WBGT) under varying temperature conditions during exercise

Supporting Table S1. Comparison of the smart-sensing lip guard and other CO_2 monitoring devices.

	Type	Feature	Multimodality	Connection	Reproduci-bility	Battery Durability	Test
**This work**	**Lip guard**	**VCO_2<br>Humidity<br>Temperature<br>Environment<br>Osmolality**	**ECG<br>Skin-Temp.<br>Accelerometer<br>Gyroscope**	**Wireless**	**O**	**Up to 13 h**	Incline: 4.8 km h^{-1}<br>Running: Up to 14.4 km h^{-1}<br>Cycling: 20 km h^{-1}
P. Grangeat (2018)[1]	Wristband	$TcPCO_2$	-	Wireless	-	6 h	Cycling (-)
S. Jimena (2021)[2]	Mask	VCO_2, VO_2	-	Wireless	-	-	-
L. Jin (2022)[3]	Mask	VCO_2, VO_2	-	Wired	O	-	Running<br>(8 km h^{-1})
V. Tipparaju (2022)[4]	Wristband	$TcPCO_2$<br>Temperature<br>Humidity	-	Wireless	X	4.5 h	-
P. Escobedo (2022)[5]	Mask	VCO_2<br>Temperature	-	Wireless	X	-	Cycling<br>(600 W)
T. Fufan (2023)[6]	Wristband	$TcPCO_2$	-	Wireless	X	-	-
H. Chen (2024)[7]	Mask	$EtCO_2$, EtO_2	-	Wired	-	-	Cycling<br>(150 W)
A. Angelucci (2024)[8]	Wristband	$TcPCO_2$	-	-	X	-	-
C. Domingo (2006)[9]	Wired Sensor	$TcPCO_2$	-	Wired	X	-	-
J. Storre (2011)[10]	Wired Sensor	$TcPCO_2$	-	Wired	X	-	-
C. Lou (2019)[11]	Mask	VCO_2	-	Wired	-	-	Cycling<br>(220 W)
B. Shykoff (2021)[12]	Mask	VCO_2	-	Wired	-	-	Cycling (-)
K. Iitani (2021)[13]	Wristband	$TcPCO_2$	-	Wired	X	-	Stepping<br>(140 spm)

Supporting Table S2. Comparison of the all-in-one smart-sensing lip guard and soft cardiac patch with other wearable athletic exercise monitoring devices.

	Type	Detection of environmental effects	Detection of CO_2	Detection of continuous salivary osmolality	Detection of cardiac signals	Activity during the device test
**This work**	**Lip guard and soft cardiac patch**	**Yes**	**Yes**	**Yes**	**ECG<br>Heartrate<br>Skin-Temp.<br>Accelerometer<br>Gyroscope**	Incline: 4.8 km h^{-1}<br>Running: Up to 14.4 km h^{-1}<br>Cycling: 20 km h^{-1}
Previous work[14]	Mouthguard and chest patch	-	-	Yes	ECG<br>Heartrate<br>Skin-Temp.<br>Accelerometer<br>Gyroscope	Running: 15–20 km h^{-1}<br>Cycling: 40 km h^{-1}
V. Tipparaju (2022)[4]	Wristband	-	Yes	-	-	-
T. Fufan (2023)[6]	Wristband	-	Yes	-	-	-
D. B. Camarillo (2013)[15]	Mouthguard	-	-	-	-	Linear impactor: 7–28 km h−1
M. Sharifuzzaman (2023)[16]	Chest patch	-	-	-	ECG	Cycling
M. Zahed (2023)[17]	Chest patch	-	-	-	ECG	Static
C. Qiu (2022)[18]	Chest patch	-	-	-	Respiration	Running: 6 km h^{-1}<br>Cycling: 20 km h^{-1}
Å. Ausland (2022)[19]	Chest patch	-	-	-	ECG, Heartrate	Running<br>Poling<br>Cycling
T. Li (2022)[20]	Chest patch	-	-	-	ECG	Cycling
T. Yamane (2022)[21]	Smart shirt	-	-	-	ECG	Running: 9 km h^{-1}
N. Faidah (2020)[22]	Handheld type device	-	-	-	-	Static
J. Aguilar-Toran (2023)[23]	Chest band	-	-	-	Heartrate	Cycling
S. Kwon (2020)[24]	Wristband	-	-	-	ECG, Heartrate	Running<br>Punching

References

1. Grangeat, P., Gharbi, S., Accensi, M. & Grateau, H. First Evaluation of a Transcutaneous Carbon Dioxide Monitoring Wristband Device during a Cardiopulmonary Exercise Test. *Ieee Eng Med Bio*, 3352-3355 (2019).
2. Mora, S.J. et al. Comparative study of a novel portable indirect calorimeter to a reference breath-by-breath instrument and its use in telemedicine settings. *Clin Nutr Espen* **46**, 361-366 (2021).
3. Jin, L. et al. Wearable Piezoelectric Airflow Transducers for Human Respiratory and Metabolic Monitoring. *Acs Sensors* (2022).
4. Tipparaju, V.V., Mora, S.J., Yu, J.J., Tsow, F. & Xian, X.J. Wearable Transcutaneous CO2 Monitor Based on Miniaturized Nondispersive Infrared Sensor. *Ieee Sens J* **21**, 17327-17334 (2021).
5. Escobedo, P. et al. Smart facemask for wireless CO2 monitoring. *Nat Commun* **13** (2022).
6. Tufan, T.B. & Guler, U. A Transcutaneous Carbon Dioxide Monitor Based on Time-Domain Dual Lifetime Referencing. *Ieee T Biomed Circ S* **17**, 795-807 (2023).
7. Chen, H.Y., Xiao, L.Y. & Guan, X.Y. Modeling and Alignment Algorithms of Multiple Sensors for the Wearable Human Respiration Monitoring System. *Ieee Sens J* **24**, 2945-2952 (2024).
8. Angelucci, A. et al. A Wearable Device to Monitor the Partial Pressure of Transcutaneous Carbon Dioxide. *Ieee T Instrum Meas* **73** (2024).
9. Domingo, C. et al. Transcutaneous measurement of partial pressure of carbon dioxide and oxygen saturation:: Validation of the SenTec monitor. *Arch Bronconeumol* **42**, 246-251 (2006).
10. Storre, J.H., Magnet, F.S., Dreher, M. & Windisch, W. Transcutaneous monitoring as a replacement for arterial CO2 monitoring during nocturnal non-invasive ventilation. *Resp Med* **105**, 143-150 (2011).
11. Lou, C.G. et al. Near-infrared tunable diode laser absorption spectroscopy-based determination of carbon dioxide in human exhaled breath. *Biomed Opt Express* **10**, 5486-5496 (2019).
12. Shykoff, B.E., Lee, L.S.R., Gallo, M. & Griswold, C.A. Transcutaneous and End-Tidal CO2 Measurements in Hypoxia and Hyperoxia. *Aerosp Med Hum Perf* **92**, 864-872 (2021).
13. Iitani, K. et al. What do masks mask? A study on transdermal CO2 monitoring. *Med Eng Phys* **98**, 50-56 (2021).
14. Kim, K.R. et al. All-in-One, Wireless, Multi-Sensor Integrated Athlete Health Monitor for Real-Time Continuous Detection of Dehydration and Physiological Stress. *Adv Sci* (2024).
15. Camarillo, D.B., Shull, P.B., Mattson, J., Shultz, R. & Garza, D. An Instrumented Mouthguard for Measuring Linear and Angular Head Impact Kinematics in American Football. *Ann Biomed Eng* **41**, 1939-1949 (2013).
16. Sharifuzzaman, M. et al. MXene/Fluoropolymer-Derived Laser-Carbonaceous All-Fibrous Nanohybrid Patch for Soft Wearable Bioelectronics. *Adv Funct Mater* **33** (2023).

17. Zahed, M.A. et al. Microfluidic-Integrated Multimodal Wearable Hybrid Patch for Wireless and Continuous Physiological Monitoring. *Acs Sensors* (2023).
18. Qiu, C.K., Wu, F., Han, W.Q. & Yuce, M.R. A Wearable Bioimpedance Chest Patch for Real-Time Ambulatory Respiratory Monitoring. *Ieee T Bio-Med Eng* **69**, 2970-2981 (2022).
19. Ausland, A., Sandberg, E.L., Jortveit, J. & Seiler, S. Heart rhythm assessment in elite endurance athletes: A better method? *Front Sports Act Liv* **4** (2022).
20. Li, T.Y. et al. An integrated and conductive hydrogel-paper patch for simultaneous sensing of Chemical-Electrophysiological signals. *Biosens Bioelectron* **198** (2022).
21. Yamane, T. et al. Trial of Sportswear Type ECG Sensor Device for Cardiac Safety Management during Marathon Running. *Adv Biomed Eng* **11**, 151-161 (2022).
22. Faidah, N. et al. Detection of voluntary dehydration in paediatric populations using non-invasive point-of-care saliva and urine testing. *J Paediatr Child H* **57**, 813-818 (2021).
23. Aguilar-Torán, J. et al. Novel Sweat-Based Wearable Device for Advanced Monitoring of Athletic Physiological Biometrics. *Sensors-Basel* **23** (2023).
24. Kwon, S. et al. Skin-conformal, soft material-enabled bioelectronic system with minimized motion artifacts for reliable health and performance monitoring of athletes. *Biosens Bioelectron* **151** (2020).
